# Thiazole Derivatives as Promising Candidates for Cryptococcosis Therapy

**DOI:** 10.1021/acsinfecdis.4c00732

**Published:** 2025-02-07

**Authors:** Victor Augusto Teixeira Leocádio, Isabela L. Miranda, Martha H. C. Magalhães, Valtair Severino dos Santos Júnior, José Eduardo Goncalves, Renata Barbosa Oliveira, Vinicius Gonçalves Maltarollo, Rafael Wesley Bastos, Gustavo Goldman, Susana Johann, Nalu Teixeira de Aguiar Peres, Daniel de Assis Santos

**Affiliations:** 1 Departamento de Microbiologia, Universidade Federal de Minas Gerais, Belo Horizonte 31270-901, Brazil; 2 Departamento de Produtos Farmacêuticos, Faculdade de Farmácia, Universidade Federal de Minas Gerais, Belo Horizonte 31270-901, Brazil; 3 Centro de Biociências, Universidade Federal do Rio Grande do Norte, Natal 59078-970, Brazil; 4 National Institute of Science and Technology in Human Pathogenic Fungi, São Paulo14040-900,Brazil; 5 Faculdade de Ciências Farmacêuticas de Ribeirão Preto, Universidade de São Paulo, Ribeirão Preto 14040-903,Brazil

**Keywords:** *Cryptococcus*, thiazole, ergosterol biosynthesis, novel antifungals, *in vivo* activity

## Abstract

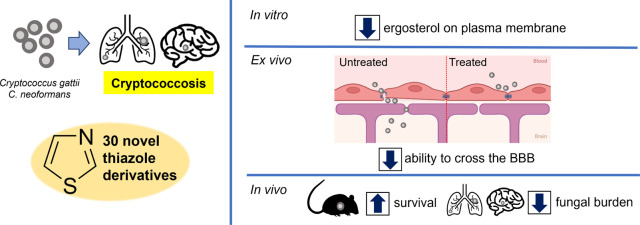

Cryptococcosis is a severe fungal infection primarily caused by two encapsulated yeasts: *Cryptococcus neoformans* and *C. gattii*. The most significant complication is cryptococcal meningitis, where the fungus crosses the blood-brain barrier, leading to a severe brain infection. Current treatments, which include amphotericin B and flucytosine or fluconazole, are often toxic and not very effective. Therefore, there is a pressing need for new antifungal agents. This study screened 30 thiazole derivatives for their antifungal activity against *Cryptococcus* and their toxicity to brain cells. Four compounds (RN86, RN88, RJ37, and RVJ42) showed particularly strong effects. These compounds reduced ergosterol levels in the fungal membrane and inhibited its ability to cross the blood-brain barrier. Notably, RN86 and RVJ42 improved survival rates in a mouse model of cryptococcosis by lowering the fungal load in the lungs and brain. These findings suggest that these derivatives could be promising treatments for pulmonary and neurocryptococcosis.

Cryptococcosis is an invasive mycosis with a high fatality rate, distributed worldwide, causing approximately 150,000 deaths per year.^1^ The most important infection caused by these species is cryptococcal meningitis, in which the fungus crosses the blood-brain barrier and causes infections in the central nervous system.^2^ Recently, there has been a decline in the number of cases due to advances in antiretroviral therapies, but the disease lethality remains significant.^3^ It is primarily caused by two encapsulated yeast species: *Cryptococcus neoformans* and *C. gattii*. Currently, the treatment for cryptococcosis involves the use of amphotericin B in combination with flucytosine and fluconazole.^4^ In addition to being costly, toxic, and prone to resistance, treatment of meningitis is more complex and less effective.^5^

The central nervous system (CNS) and lungs are the organs most affected by cryptococcal infections. Lung infection can manifest as asymptomatic colonization or as noncalcified nodules and pulmonary infiltrations.^6^ In immunocompromised individuals, the most common clinical manifestation is a pneumonia that quickly progresses to acute respiratory distress syndrome, a condition seen in more than 50% of AIDS patients who die from fungal pneumonia.^7^ CNS infections present with symptoms such as fever, headaches, meningismus, papilledema, and even seizures.^8^

The strategy for the treatment of cryptococcosis is directly linked to the location and severity of the infection and the host’s immune status.^9^ The search for new antifungal agents is a broad field, but only a few drugs are approved by regulatory agencies and used in disease treatment.^10^ Azoles are a class of heterocyclic chemical compounds that contain a nitrogen atom and at least one other noncarbon atom as part of the ring, used since the 1950s as antifungals.^11^ Thiazoles have a sulfur atom and another nitrogen atom as the other noncarbon atom, with a molecular formula of C_3_H_3_NS. Synthetic thiazoles have shown promising anti-inflammatory and antimicrobial activities, resulting in great interest from a pharmaceutical perspective.^12,13^ The effect of some thiazoles on *Cryptococcus* spp. has been previously demonstrated *in vitro*, leading to increased intracellular oxidative stress^14^ and influencing fungal virulence, causing a decreased biofilm formation and capsule thickness.^15^ Therefore, thiazole derivatives show promising antifungal activity and can be studied for their ability to cross and interfere with the blood-brain barrier to treat cryptococcosis. In this study, we tested a series of 30 thiazole derivatives with antifungal activity, some of which were already described^13,16−18^ for their *in vitro* activity against *Cryptococcus* spp. The most active compounds were further studied regarding their mechanism of action, ability to cross the blood-brain barrier, and *in vivo* activity in a murine model.

## Results

### Antifungal Activity of Thiazole Derivatives against Cryptococcus spp

Initially, 30 thiazole derivatives were tested against *C. neoformans* H99 and *C. gattii* R265 strains to screen the compounds presenting the best antifungal activity. The lowest values of minimum inhibitory concentrations (MICs) were 0.2 and 0.1 μg/mL for derivatives RJ37 and RVJ42, respectively. The highest MIC values were obtained for RN18, RN99, RN100, and RVJ37, with MICs of 12.5, 12.5, and higher than 50 μg/mL, respectively (Table 1). After the MIC test, MFC values were also obtained. For all the substances tested, the MFC/MIC ratio was 1, indicating a possible fungicidal action of the compounds tested.^19^

**Table 1:** Minimum Inhibitory Concentration (MIC) of the 30 Thiazole Derivates against *C. neoformans* H99 and *C. gattii* R265 Strains, Values of Cytotoxicity Index (CI_50_) in the hCMEC/D3 Cell Line, and the Selectivity Index for 12 Thiazole Derivatives and Fluconazole

	**minimum inhibitory concentration (μg/mL)**				
**compound**	*C. neoformans***H99**	*C. gattii***R265**	**CI**_**50**_**(μg/mL)**	**selectivity index**	**reference**
RN18	6.3	12.5	>300	32	(20)
RI20	6.3	6.3	9.4	1.5	(13)
RN40	1.6	0.8	150	128.2	(13)
RN86	3.1	1.6	150	64	(21)
RN88	1.6	0.8	37.5	31.6	(21)
RN99	12.5	12.5	4.7	0.4	(13)
RN100	25	12.5	37.5	2	(13)
RN104	0.8	0.8	2.3	2.9	(13)
RN105	0.8	0.8	18.8	24.1	(13)
RN112	3.1	1.6	2.3	1	(13)
RN113	0.8	0.8			(13)
RJ13	1.6	0.8			(16)
RJ30	6.3	6.3			(17)
RVJ31	6.3	50			(17)
RVJ33	3.1	1.6			(17)
RVJ35	1.6	1.6			(17)
RJ35	0.2	<0.1			(17)
RVJ36	0.8	0.8			(17)
RVJ37	>50	>50			(17)
RJ37	0.2	0.2	150	750	(17)
RJ38	0.4	0.2			(17)
RVJ42	0.2	0.1	75	500	(17)
RJ44	0.8	0.8			(17)
RVJ45	0.4	0.2			
RVJ46	12.5	12.5			(19)
RVJ47	0.4	0.4			(22)
RJV49	0.2	0.4			(22)
RJ32	1.6	0.8			(17)
RVJ62	3.1	3.1			(18)
PD76	0.8	0.4			(19)
amphotericin B	0.4	0.4			
fluconazole	2.0	4.0	>300	96.5	

### Thiazole Derivatives Are Nontoxic to Blood-Brain Barrier Cells

**Figure 1:**
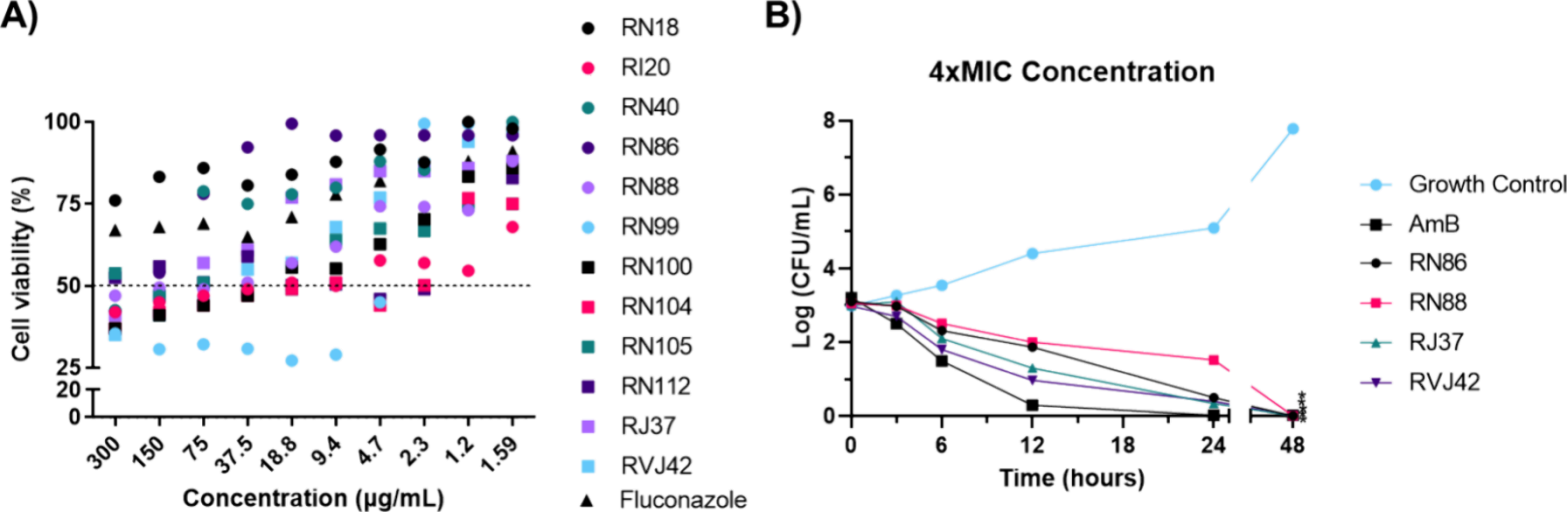
Thiazoles are nontoxic to hCMEC/D3 cells and inhibit *Cryptococcus neoformans* growth. (A) Percentage of cell viability of hCMEC/D3 growth at different thiazoles concentrations. (B) Effect of different thiazoles concentrations on the growth of *Cryptococcus neoformans* H99 strain. Statistical analysis: area under the curve; one-way ANOVA followed by the Tukey post-test. * *p* < 0.05 compared to growth control. MIC: minimum inhibitory concentration.

The toxicity of the compounds for the hCMEC/D3 cell line was initially verified through a cell viability assay using the 12 most effective compounds against *Cryptococcus* spp. (those that had the lowest MIC values) (Figure 1A). Of the 12 thiazoles tested, RN20, RN99, RN100, RN112, and RN113 presented a selectivity index value (SI = IC_50_/MIC) lower than 10, indicating possible toxicity to nontarget cells, while the RJ37 and RVJ42 molecules presented the highest SI values, indicating the absence of toxicity (Table 1).

### Fungal Growth Kinetics

Next, a time–kill curve experiment was carried out in the presence of the four least toxic and most active compounds (RJ37, RVJ42, RN86, and RN88), using the MIC, 2 × MIC, and 4 × MIC concentrations for the H99 strain (Figure 1B, Figures S14 and S15). For the 2 × MIC concentration, after 24 h of incubation, all substances showed a reduction of approximately 1.5log, except for RN88, which was reduced by 1log. After 48 h, the compounds had a reduction of 3log, while RN86 had a reduction of 1.5log. At the 4 × MIC concentration, after 24 h of incubation, all substances showed a reduction of approximately 2.5log, except for RN88, which was reduced by 1.5log. After 48 h, all compounds had a reduction of 3log.

Then, these four compounds were tested for 11 strains of *C. neoformans* and *C. gattii*. MIC values obtained after expanding the number of strains remained similar to those observed with the two initially used in the screening, with the MIC varying from 0.1 to 1.6 μg/mL (Table 2).

**Table 2:** Minimum Inhibitory Concentration of Four Thiazoles and Fluconazole against 11 *C. neoformans* and *C. gattii* Strains^a^

	**minimum inhibitory concentration (μg/mL)**					
**strains**		**compounds**				
		**RJ37**	**RVJ42**	**RN86**	**RN88**	**FLZ**
*C. neoformans*	ATCC 24067	0.2	0.1	0.8	0.4	2.0
	WM 629 (ATCC MYA-4567)	0.8	0.2	1.6	0.4	4.0
	WM 626 (ATCC MYA-4565)	0.4	0.4	3.1	1.6	2.0
	WM 628 (ATCC MYA-4566)	0.4	0.2	<0.1	<0.1	4.0
	ATCC 28957	0.4	0.1	0.8	0.4	2.0
	geometric mean	0.4	0.2	1.3	0.5	2.8
*C. gattii*	L27/01	0.4	0.2	0.8	0.4	2.0
	29/0893	0.4	0.8	1.6	1.6	2.0
	547 OTTI	0.2	0.2	0.8	0.4	4.0
	96806	0.2	0.4	1.6	1.6	4.0
	28/0893	0.4	0.1	0.8	0.8	4.0
	1962	0.4	0.2	1.6	0.4	2.0
	geometric mean	0.3	0.3	1.2	0.9	3.0

a FLZ: fluconazole.

### Knockout Strains for Transcription Factors Involved in the Response to Cell Stress Are Resistant to Thiazole Derivatives

**Figure 2:**
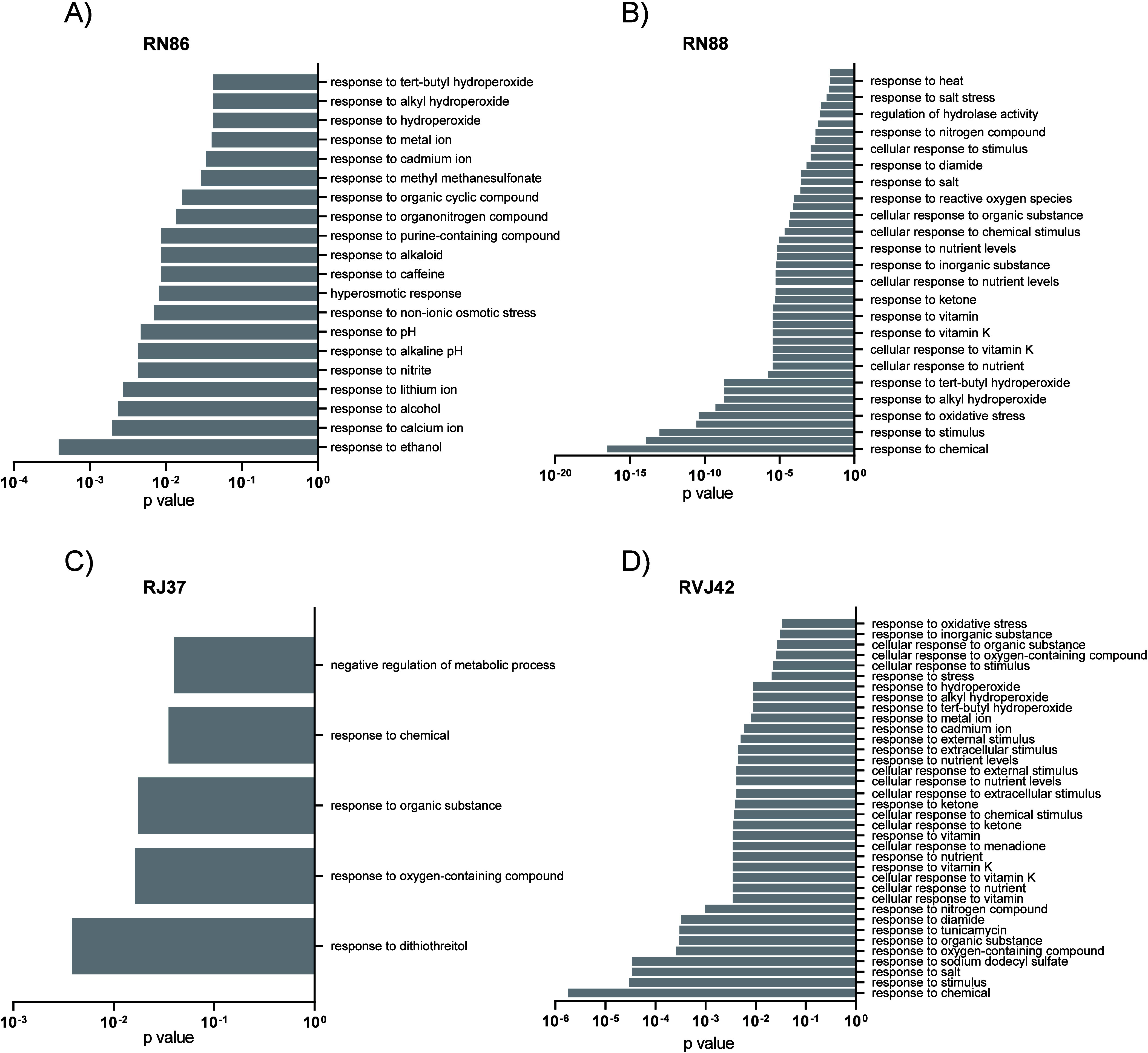
Functional categorization of the *C. neoformans* H99 transcription factors (TF) deletion strains resistant to the thiazole derivatives. (A) H99 TF mutants resistant to RN86. (B) H99 TF mutants resistant to RN88; (C) H99 TF mutants resistant to RJ37. (D) H99 TF mutants resistant to RVJ42. Gene Ontology (GO) terms for the analysis of biological processes related to responses to various stresses have statistically significant *p*-values. The concentrations used in this experiment for compounds RN86, RN88, RJ37, and RVJ42 were 12.4, 6.4, 0.8, and 0.8 μg/mL, respectively. Statistical analysis: *p*-value from Fisher’s exact test.

The chemical-genetic screening using the *C. neoformans* H99 transcription factors deletion library^23^ revealed several mutants resistant (MIC for thiazoles higher than 4× the original MIC value) to thiazoles: two to RN86, 19 to RN88, four to RVJ37, and seven to RJ42 (Table 3). Gene Ontology (GO) functional categorization of these transcription factors showed that they are involved in biological processes and molecular functions (data not shown) in responses to osmotic and oxidative stress and DTT, and also in melanin biosynthesis (Figure 2). Resistant mutants are associated with capsular enlargement (*ZAP104*, *ADA2, RDS2, USV101*, and *SKN7*), resistance to high salt concentrations (*SKN7* and *NRG1*), increased melanin production (*USV101* and *SKN7*), resistance to several compounds such as heavy metals (*ADA2*, *RDS2*, and *ZNF2*), tunicamycin (*PPR1*, *HEL2*, and *ZFC2*), and fluconazole (*LIV1* and *JJJ1*).^23^ These results indicate that these thiazoles trigger several pathways in response to stress and also suggest that these transcription factors regulate genes that may be the molecular targets for thiazoles.

**Table 3:** *C. neoformans* Transcription Factor Involved in Sensitivity to Thiazole Derivatives

**compound**	**gene ID**	**gene**	**deleted gene product**
RN86	CNAG_05392	*ZAP104*	specific RNA polymerase II transcription factor
	CNAG_01626	*ADA2*	transcriptional adapter 2-alpha
RN88	CNAG_02296	*RBK1*	ATP-binding protein
	CNAG_05785	*STB4*	putative transcription factor
	CNAG_06425	*PPR1*	fungal-specific transcription factor
	CNAG_07924	*MCM1*	pheromone receptor transcription factor, putative
	CNAG_00896	*FZC34*	transcription factor
	CNAG_06814	*SXI1alpha*	alpha cell-type homeodomain transcription factor
	CNAG_01454	*STE12alpha*	transcription factor STE12
	CNAG_03527	*HEL2*	cytoplasmic protein
	CNAG_04268	*APN2*	exodeoxyribonuclease III
	CNAG_05420	*USV101*	nutrient and stress factor 1, putative
	CNAG_03409	*SKN7*	osmolarity two-component system, response regulator SKN7
	CNAG_00460	*LIV1*	virulence related protein of unknown function
	CNAG_03902	*RDS2*	regulator of drug sensitivity 2, putative
	CNAG_04093	*YRM103*	putative transcription factor
	CNAG_03894	*PDR802*	putative Zn2-Cys6 zinc-finger transcription factor
	CNAG_03018	*ASG101*	putative zinc finger transcription factor
	CNAG_01973	*ZFC2*	C2H2 zinc finger protein Zas1A
	CNAG_07922	*FZC4*	transcription factor
	CNAG_03366	*ZNF2*	C2H2 type zinc finger transcription factor
RJ37	CNAG_00193	*GAT1*	GATA type zinc finger protein asd-4
	CNAG_02774	*MAL13*	fungal Zn(2)-Cys(6) binuclear cluster domain protein
	CNAG_01173	*LAG1*	LAG1 family transcription factor, putative
	CNAG_05785	*STB4*	putative transcription factor
RVJ42	CNAG_07506	*FAP1*	FKBP12-associated protein 1, putative
	CNAG_07797	*CLR6*	transcriptional regulator, variant
	CNAG_05222	*NRG1*	transcriptional regulator Nrg1
	CNAG_01173	*LAG1*	LAG1 family transcription factor, putative
	CNAG_06064	*PTP1*	putative protein tyrosine phosphatase
	CNAG_06425	*PPR1*	fungal specific transcription factor
	CNAG_05538	*JJJ1*	DnaJ-like cochaperone

### Exposure to Thiazole Derivatives Increases Fungal Growth under Stressing Conditions and Alter the Production of Melanin

**Figure 3:**
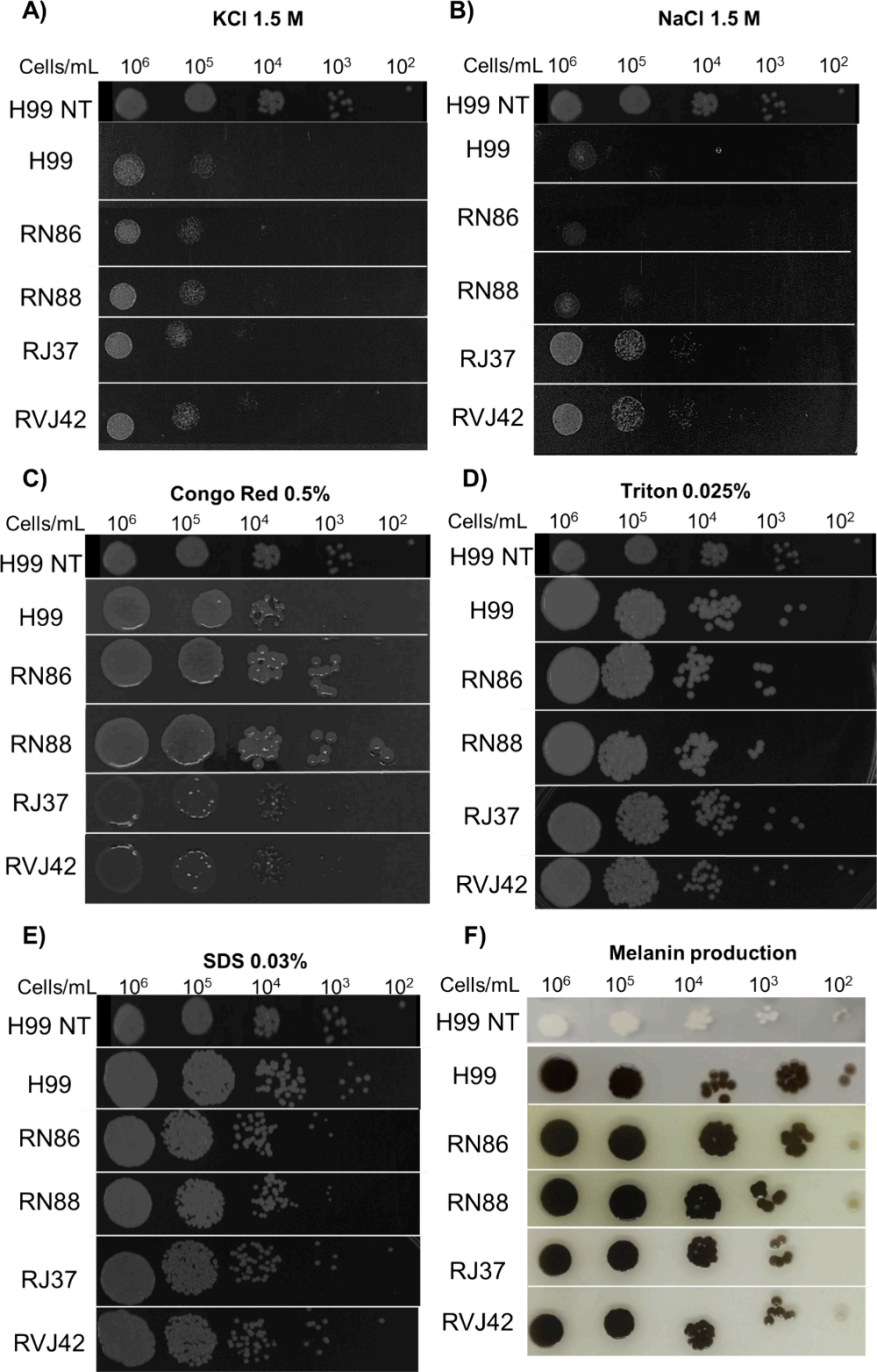
Growth of *C. neoformans* H99 under stress conditions after exposure to thiazole derivatives in subinhibitory concentrations, using a serial dilution of 1 × 10^6^ to 1 × 10^2^ cells/mL. H99 NT indicates growth control in SDA without stress or exposure to thiazole derivatives. H99 indicates the growth control without exposure to thiazole derivatives. (A) H99 growth in KCl after exposure to thiazole derivatives. (B) H99 growth profile in NaCl after exposure to thiazole derivatives. (C) H99 growth profile in Congo Red after exposure to thiazole derivatives. (D) H99 growth profile in Triton after exposure to thiazole derivatives. (E) H99 growth profile in SDS after exposure to thiazole derivatives. (F) Growth profile of the H99 strain in minimal medium supplemented with L-DOPA after exposure to thiazole derivatives. The concentrations used in this experiment for compounds RN86, RN88, RJ37, and RVJ42 were 1.55, 0.8, 0.1, and 0.1 μg/mL, respectively.

Considering the data from the chemical-genetic screening, which pointed to the resistance to several stressors, we evaluated the growth of H99 under several stressing conditions after exposure at subinhibitory doses of the four most active thiazoles and compared it with the growth of H99 without exposure (H99 NT). We observed that thiazole pre-exposure increased fungal growth in media containing osmotic (sodium chloride and potassium chloride) (Figure 3A,B) and cell wall stressors (Congo red) (Figure 3C). In medium containing cell membrane stressor (SDS) (Figure 3E), only H99 pre-exposed to RJ37 and RVJ42 showed increased growth. As for membrane and cell wall stress (Triton X-100) (Figure 3D), only the pre-exposure to RVJ42 stood out with increased growth (Figure 3). Melanin production was reduced when the H99 strain was previously exposed to thiazoles (Figure 3F). It is possible to see a change in the phenotype of the strains exposed to the derivatives, with a lighter color compared to the control without exposure. These findings suggest that pre-exposure to the thiazole derivatives may generate cellular changes and increase fitness under stress conditions.

### Antagonism of Thiazoles with Fluconazole and Amphotericin B Points to the Ergosterol Pathway as the Probable Thiazole Target

**Figure 4:**
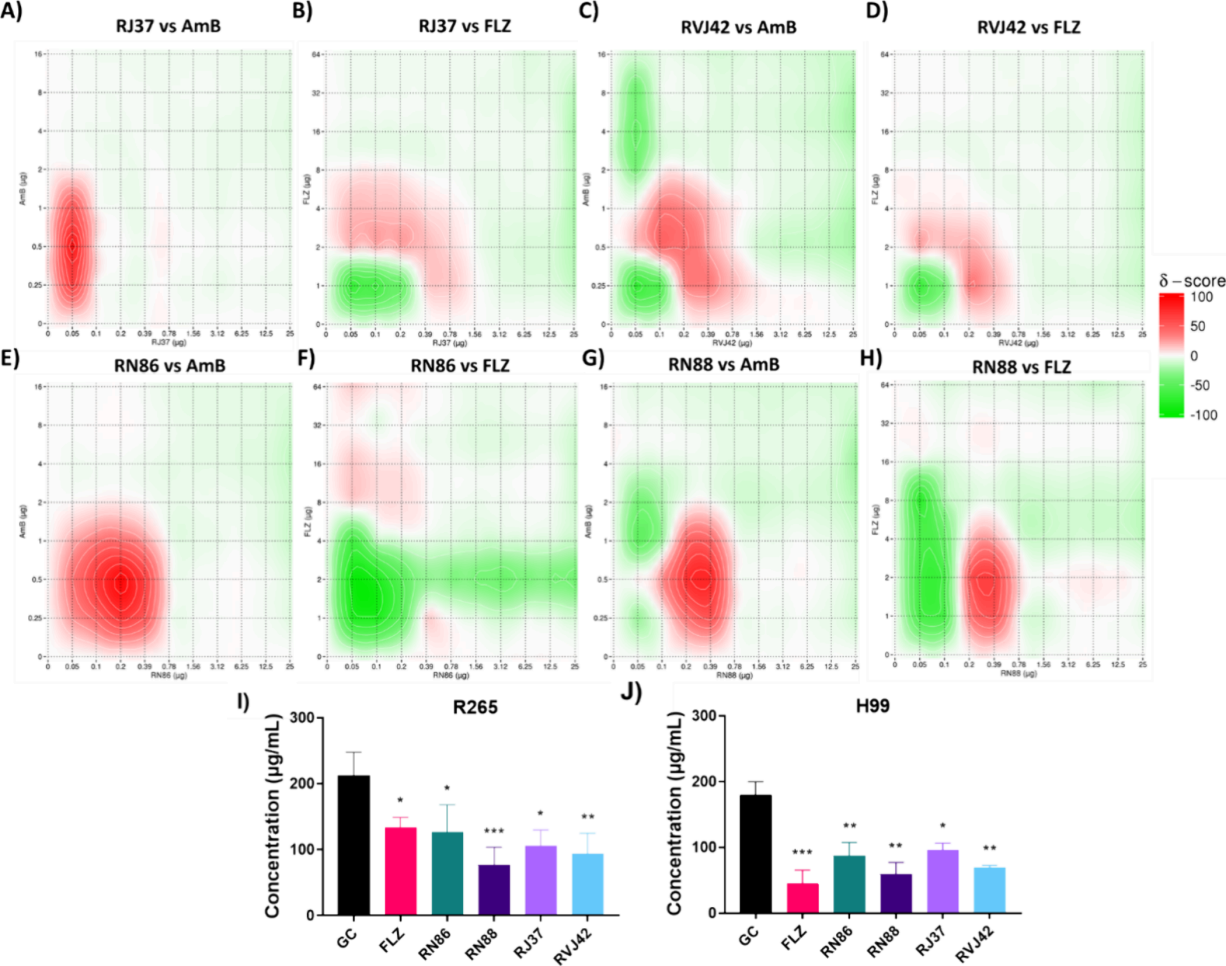
Interaction between thiazole derivatives and amphotericin B or fluconazole and the production of ergosterol in *Cryptococcus* strains after exposure to subinhibitory concentrations of the thiazole derivatives. (A) RJ37 versus AmB; (B) RJ37 versus FLZ; (C) RVJ42 versus AmB; (D) RVJ42 versus FLZ; (E) RN86 versus AmB; (F) RN86 versus FLZ; (G) RN88 versus AmB; (H) RN88 versus FLZ. Red indicates a synergistic interaction, while green indicates antagonism. (I, J) Ergosterol content of the *C. neoformans* H99 (I) and *C. gattii* R265 (J) strains after treatment with subinhibitory concentrations of fluconazole or thiazole derivatives. The concentrations used in this experiment for compounds RN86, RN88, RJ37, and RVJ42 were 1.55, 0.8, 0.1, and 0.1 μg/mL, respectively. FLC: fluconazole; GC: growth control. (A–H) Graphics generated using the SynergyFinder 3.0 platform with the zero interaction potency (ZIP) model. (I, J) Statistical analysis: one-way ANOVA followed by the Tukey post-test, in relation to growth control. * *p* < 0.05, ** *p* < 0.005, *** *p* < 0.0005.

The interaction between thiazole derivatives and clinical antifungals, determined by the zero interaction potency (ZIP) model, proved to be dose-dependent. The ZIP model analyzes drug interaction relationships by comparing the changes in potency of the dose–response curves for individual drugs and their combinations. The ZIP score (δ) generated by the interaction between thiazoles with fluconazole or AmB showed that antagonism (δ < 0) occurs when the two drugs are at their highest concentrations: between 3.12 and 25.0 μg/mL for thiazoles and between 2 and 64 μg/mL for fluconazole and 1 and 16 μg/mL for AmB (upper right portions of the graphs). The synergistic interaction (δ > 0) was observed only for 0.05 to 0.78 μg/mL of thiazole derivatives, 1 to 8 μg/mL of fluconazole, and 0.25 to 2 μg/mL of AmB (Figures 4A-H).

In addition to the antagonistic interaction, the thiazole derivatives reduced the production of ergosterol for the H99 and R265 strains, with an average reduction of 43%, similar to fluconazole (Figure 4I,J).

### Thiazoles Cross the BBB and Reduce Fungal Load

**Figure 5:**
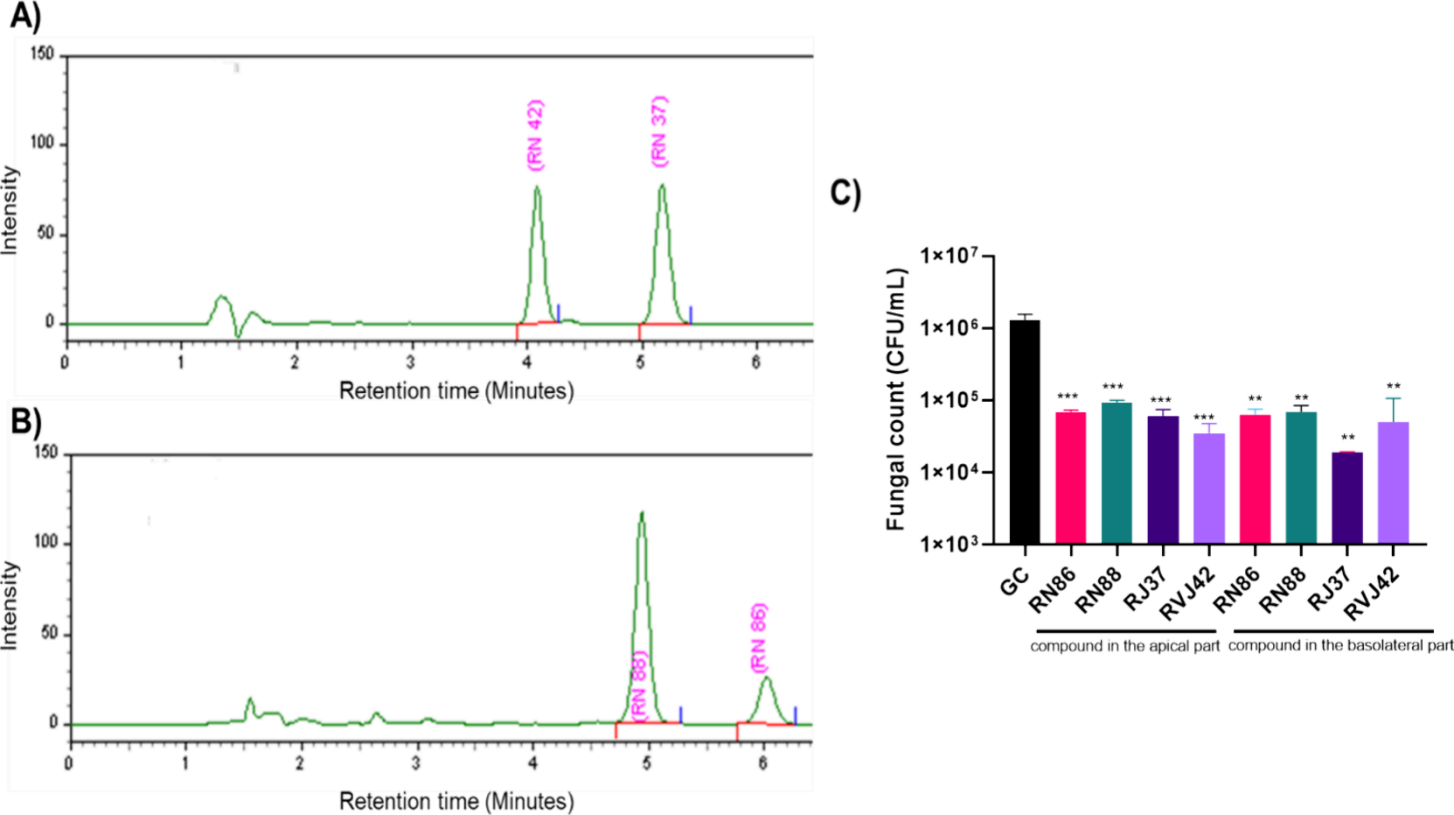
Chromatograms of thiazoles and fungal load obtained after the transcytosis assay in the in vitro BBB model using the hCMEC/D3 cell line. (A) Chromatogram generated in the analysis of compounds RJ37 and RVJ42 at 20 μg/mL under the optimized conditions of the chromatographic method. (B) Chromatogram generated in the analysis of compounds RN86 and RN88 at 20 μg/mL under the optimized conditions of the chromatographic method. Compounds RJ37, RVJ42, RN86, and RN88 have their release peaks at 5.2, 4.1, 6.0, and 4.9 min, respectively. (C) Transcytosis assay of the *C. neoformans* H99 strain in the hCMEC/D3 cell line treated with one of the RN86, RN88, RJ37, and RVJ42 derivatives. H99 were placed in the basolateral part and thiazoles were placed either in the apical or basolateral parts. The concentrations used in this experiment for compounds RN86, RN88, RJ37, and RVJ42 was 3.1, 1.6, 0.2, and 0.2 μg/mL, respectively. GC: Growth control. Statistical analysis: one-way ANOVA with the Tukey post-test. * *p* < 0.05, ** *p* < 0.005, *** *p* < 0.0005.

Considering that *Cryptococcus* spp. causes meningoencephalitis, it is crucial that thiazoles cross the BBB to treat this disease. Using an *in vitro* BBB model, we quantified the thiazoles in the basolateral of the transwell, where BBB was performed. Starting the biological sample quantification stage, the chromatogram generated for each of the tested compounds presents free peaks with distinct and well-defined retention times, showing the good selectivity of the quantification methodology (Figure 5A,B). Following the quantification step, the analysis of the biological samples after the compound passed through the hCMEC/D3 monolayer can be seen in Table 4. For all four molecules, a higher percentage of the initial concentration was observed in the experiments using 20 μg/mL of the derivatives. The average recovered for the concentration of 20 μg/mL was 4.2%, while for the concentrations of 200 μg/mL, it was 0.9%.

**Table 4:** Concentration of the Compounds Added to the Blood Brain Barrier Model in a Transwell System in the Apical Part and Then Recovered from the Basolateral Part

**compound**	**concentration added (μg/mL)**	**concentration recovered (μg/mL)**	**percentage**
RN86	20	1.54	7.7
	200	1.69	0.8
RN88	20	1.03	5.2
	200	4.30	2.2
RJ37	20	0.58	2.9
	200	0.93	0.5
RVJ42	20	0.21	1.1
	200	0.43	0.2

When we infected the BBB model with *C. neoformans*, simulating the treatment with thiazole present in either the apical or basolateral part of the system, their fungicidal activity was verified by comparing the fungal load of untreated controls. In the growth control, a concentration of 1.3 × 10^6^ CFU/mL and an average of 5.6 × 10^4^ CFU/mL were obtained for yeast in the wells where the derivatives were placed in the apical part, and 5.7 × 10^4^ CFU/mL in the wells where the derivatives were placed in the basolateral part (Figure 5C). Moreover, it was noted that the compounds added to the upper apical compartment could directly target the fungi already present in the basolateral compartment. These findings suggest that the compounds have the ability to penetrate the BBB and kill the fungus.

### Lethality and Fungal Burden Are Reduced in Mice Treated with Thiazoles

**Figure 6:**
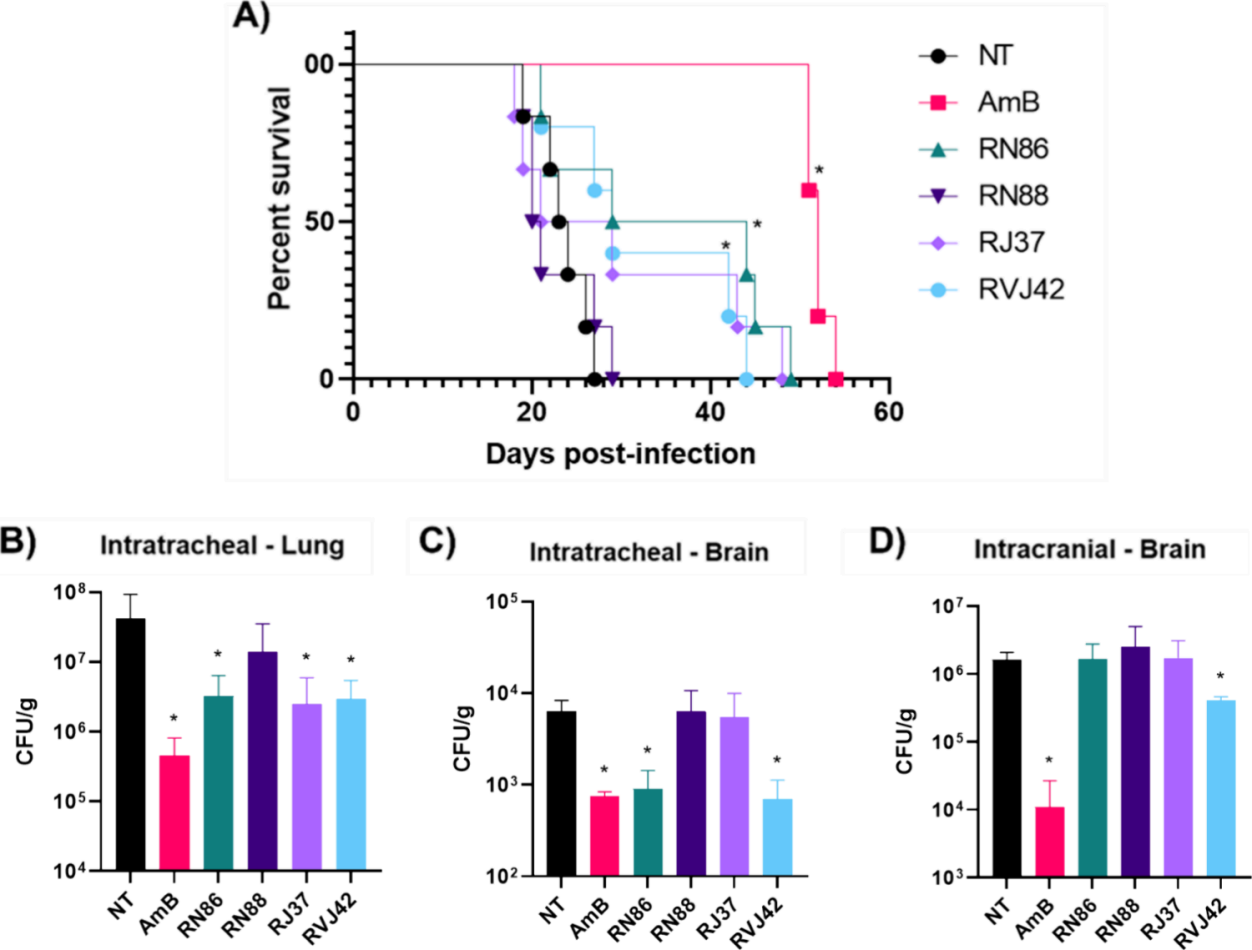
Survival and fungal burden of mice infected with the H99 strain and treated or not with thiazoles. (A) Lethality curve of mice infected with the H99 strain after treatment with thiazoles and Amphotericin B (AmB). (B, C) Fungal load in the brain and lung after 10 days of intratracheal infection with 1 × 10^5^*C. neoformans* H99 cells and daily treatment with amphotericin B or thiazole derivatives. (D) Fungal load obtained in the brain after 5 days of intracranial infection with 1 × 10^3^*C. neoformans* H99 cells and daily treatment with amphotericin B or thiazoles. NT: untreated group. Survival curve was plotted by the Kaplan–Meier method, and the results were analyzed using the log rank test, # *p* < 0.05 compared to NT, * *p* < 0.05 compared to AmB alone.

Finally, we evaluated the effects of thiazoles on murine cryptococcosis. The average survival was 24 days for the untreated group and 52 days for the group treated with AmB. Groups treated with RJ37 and RN88 did not have reduced lethality, presenting average survivals of 25 and 21 days, respectively. Treatments with RN86 and RVJ42 increased lifespan, with an average survival of 37 and 29 days, respectively (Figure 6A).

When analyzing the organs of the groups that underwent intratracheal infection, the fungal load for the groups treated with AmB, RN86, RJ37, and RVJ42 is statistically lower compared to the NT group (Figure 6B). The average fungal load in the lungs in the NT group was 4.2 × 10^7^ CFU/g. For the treated groups, there was a decrease in fungal load, averages of 4.5 × 10^5^, 3.2 × 10^6^, 2.5 × 10^6^, and 2.9 × 10^6^ CFU/g for AmB, RN86, RJ37, and RVJ42, respectively. Further, fungal load also decreased in the brain (Figure 6C) of the groups that received AmB, RN86, and RVJ42, compared to the NT group. The average fungal load recovered in the NT animals was 6.3 × 10^3^ CFU/g. For the treated groups, the averages were 7.5 × 10^2^, 8.9 × 10^2^, and 6.9 × 10^2^ CFU/g for AmB, RN86, and RVJ42, respectively.

In the groups infected intracranially (Figure 6D), the fungal load in the brain of the animals receiving AmB was lower, compared to untreated animals, 1.09 × 10^4^ and 1.61 × 10^6^ CFU/g, respectively. Animals that received treatment with thiazole derivatives had an average fungal load in the brain of 1.55 × 10^6^ CFU/g. Animals treated with RVJ42 had a reduction in fungal load, compared to the untreated control, being the most effective compound tested.

## Discussion

As a neglected disease, it is difficult to obtain absolute numbers for cryptococcosis, but calculations and estimates pointed to around 950,000 annual cases of cryptococcal meningitis around the world before antiretroviral therapy. After the inclusion of modern antiretroviral therapy, this number fell considerably to a total of almost 150,000 cases per year.^1^ The most commonly observed fatal form of the disease is cryptococcal meningitis as an opportunistic disease in immunocompromised HIV-positive individuals.^24^

Therapeutical options are limited, and the development of novel antifungal agents is crucial. Here, we tested a series of thiazole derivatives regarding their anticryptococcal activity. In vitro assays showed that the fungal strains analyzed were inhibited and the drugs showed a fungicidal effect, similar to amphotericin B, with no cytotoxic effect in the HCMEC cell line. Inhibition increased with increasing concentrations tested until growth completely ceased. Selected drugs with a higher potential for treatment were further evaluated to identify possible molecular targets, interaction with the yeast’s cellular transmigration capacity, ability to trans-pass cell barriers, combinatory effect with AmB and fluconazole in vitro, and in vivo treatment.

Chemical-genetic screening is an important assay that allows a broad-spectrum view of how a drug acts in a cell. By analyzing *C. neoformans* deletion mutants for several transcription factors^23^ that were resistant to the thiazole tested, it was possible to identify those genes involved in the susceptibility to these compounds, allowing us to understand the mechanisms of action of the drugs. Among the thiazole-resistant strains, mutants with increased capsular thickness were observed, which may be directly related to an increase in resistance to various drugs, with the capsule being the first physical barrier that compounds encounter when interacting with the yeast.^25^ We also observed genes involved in resistance to heavy metals, which may indicate an increase in the presence of ATPases as extrusion pumps to eliminate ions and antifungals from the interior of the cell.^26^ Moreover, mutants resistant to ER stress may be closely linked to the mechanism of action of the thiazole derivatives. Since the biosynthesis of ergosterol and several other yeast lipids occurs in the ER.^27^ Drugs acting on this synthesis can cause damage to this organelle and hinder its functioning.^28^ Higher resistance to ER stress may also be linked to greater resistance to compounds that act in lipid biosynthesis. Finally, genes that confer resistance to fluconazole were also observed. Resistance to fluconazole is normally linked to higher expression of efflux pumps and mutation or hyperexpression of the ERG11 gene, responsible for the production of the molecular target of this drug.^29^ This resistance to this azole may be directly linked to the effective mechanism of action of thiazoles. Furthermore, these transcription factors may regulate the expression of genes that are important for thiazole action in *C. neoformans*, which may also be their molecular targets.

In addition, the direct decrease in ergosterol content after treatment supports the hypothesis of the action of thiazoles on sterols, as well as the antagonism with fluconazole and amphotericin B. One hypothesis is that the combined interaction of thiazoles and polyenes leads to antagonism is that thiazoles reduce the amount of ergosterol present in the cell membrane, reducing the targets of polyenes.^30^ This effect has also been observed in a study by our group,^31^ where the antagonism of the combination of fluconazole and amphotericin B in vitro was dose-dependent and observed for high concentrations of fluconazole in *C. gattii*. In addition to the results found here on the production of ergosterol and the combinatory effect of the drugs, previous *in silico* analyses by molecular docking^32^ pointed out the enzyme lanosterol 14α-demethylase (CYP51) as a target for thiazoles.

The exposure of yeast to thiazoles decreased the recovery of H99 in the basolateral part of the BBB model, demonstrating the effective action of these compounds *in vitro*. The permeability of the molecules was demonstrated once their fungicidal activity was observed when placed in different compartments of the BBB model and by chromatography analysis. The greatest difficulty in treating diseases in the CNS is finding drugs that can cross the BBB, overcoming the strong connections in the occlusion zone and the highly active efflux pumps. Around 98% of molecules targeting the CNS cannot be used due to the low concentration found in this site.^33^ This low permeability was also observed in this study through data obtained from HPLC/DAD analyses. One way to overcome this problem is to increase the permeability of the molecules, such as the use of liposomes carrying antibodies to receptors present in BBB cells^34^ and LDL receptors.^35^

The *in vivo* studies demonstrated the antifungal action of the compounds under analysis, given the increased survival of mice infected with cryptococcosis and treated and the decreased fungal load recovered. The efficiency of each derivative *in vivo* corroborates the results obtained *in vitro*, showing RVJ42 as the most effective and promising thiazole for cryptococcosis therapy. Although RVJ42 had the lowest transmigration percentage in the BBB model, the *in vivo* activity in mice infected with the brain appears to be higher than those of the other thiazoles, corroborating MIC values.

In conclusion, the compound RVJ42 may be promising for treating cryptococcal meningitis.

## Materials and Methods

### Antimicrobial Agents

**Figure 7:**
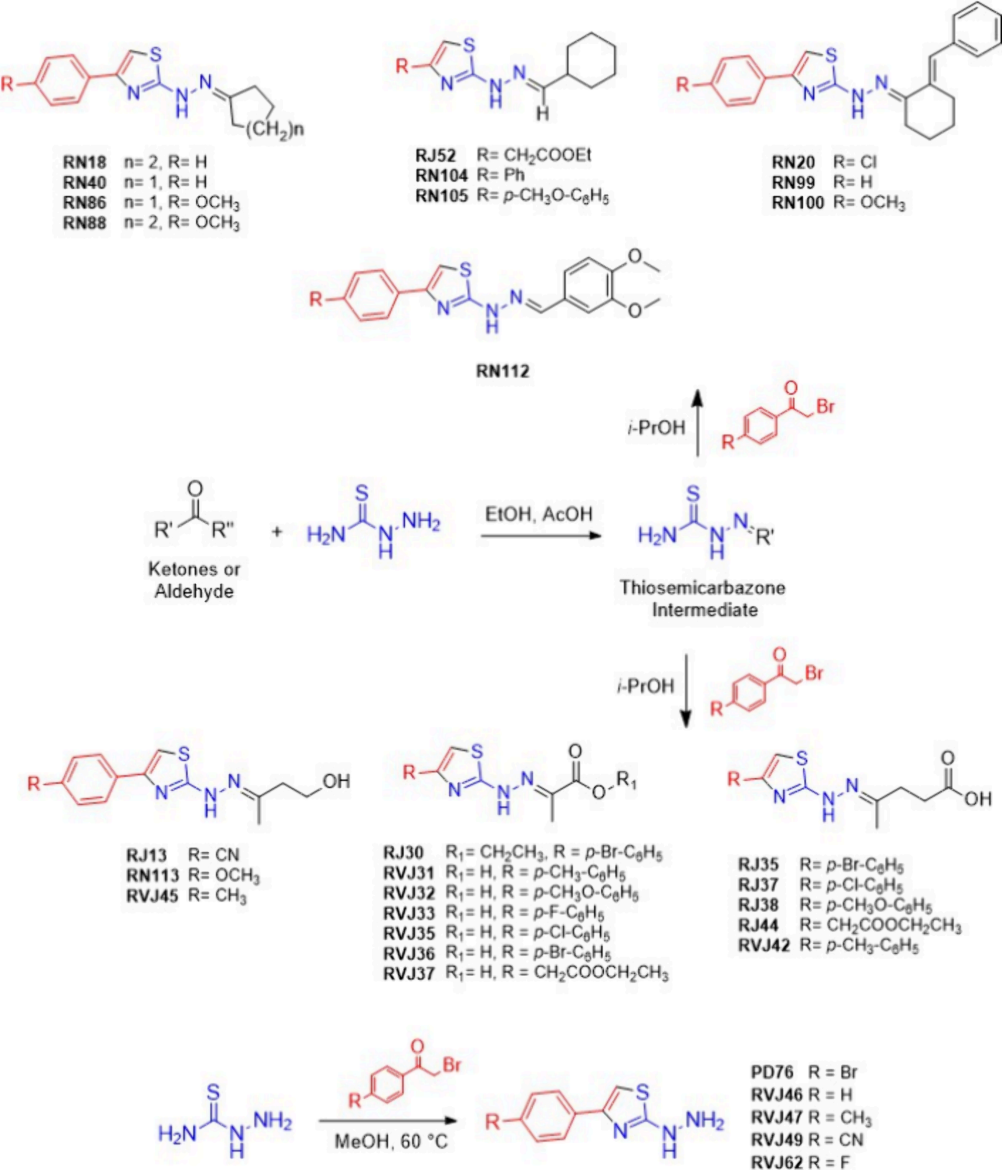
General synthesis scheme and chemical structures of the compounds: moiety derived from aldehyde or ketones in black; moiety derived from thiosemicarbazide in blue, and moiety derived from 2-bromoacetophenone in red.

Thirty thiazoles were evaluated for their antimicrobial activity against *Cryptococcus* sp. (Figure 7). Twenty-three of the 2-thiazolylhydrazones and the 2-thiazolylhidrazines PD76 were previously synthesized by our group,^16−18,20^ and the synthesis of other 2-thiazolylhidrazines RVJ46, RVJ47, RVJ49, and RVJ62 has already been described by other authors.^22,36,37^ Fluconazole and amphotericin B (Sigma-Aldrich) were commercially acquired and used as positive controls, being solubilized as recommended by the manufacturer.

#### General Experimental Procedures

The solvents used were of analytical grade and purchased from Sigma-Aldrich. Analytical thin-layer chromatography (TLC) was performed using precoated 0.5 mm plates with a suspension formed by 8 g of silica gel 60 with CaSO_4_ 13% m/m (Sigma-Aldrich) in 19 mL of distilled water. The spots were visualized with iodine vapor followed by heating at 90 °C. Compounds were fully characterized by their melting points and nuclear magnetic resonance (NMR) spectra. Melting points were measured on a Microqumica MQAPF-301 apparatus and were not corrected. UHPLC-HRMS/MS analyses were performed on a Nexera UHPLC-system (Shimadzu) hyphenated to a maXis high-resolution ESI-QTOF mass spectrometer (Bruker) and operated with the Compass 1.7 software package (Bruker). NMR spectra were recorded on Bruker AVANCE III HD OneBay (400 MHz) and Bruker AVANCE Neo Ascend (600 MHz) spectrometers and measured at a temperature of 300.15 K. Tetramethylsilane (TMS) was used as an internal standard. Chemical shifts are expressed in δ (ppm) scale and *J* values are given in Hz. The purity of the final compounds was 95% or higher and the data for the four lead derivatives (RJ37, RVJ42, RN86, and RN88) have already been described previously.^17,21^

#### Synthesis of Thiazole Derivatives

##### Synthesis of (*E*)-3-(2-(4-Methylfenil)thiazol-2-yl)hydrazinelylidene)butan-1-ol (RVJ45)

A mixture of 1 equiv of the 2-(4-hydroxybutan-2-ylidene)hydrazine-1-carbothioamide (0.31 mmol) and 1 equiv of the 2-bromo-4′-methylacetophenone (0.31 mmol) in 5 mL of 2-propanol was stirred at 80 °C for 2 h. At the end of the reaction, the solvent was removed under reduced pressure and 20 g of crushed ice and 10 mL of aqueous solution of NaHCO_3_ (10% m/v) was added to the residue obtained. The material was filtered in vacuum using a Büchner funnel. The final solid residue was washed with cold diethyl ether (7 mL), forming a suspension. The supernatant was collected using a pipet, and the resulting solid was dried under vacuum to afford RVJ45 as a brown amorphous solid (68.6 mg, 80% yield). mp: 177–178 °C; ^1^H NMR (600 MHz, DMSO-*d*_6_): δ 7.74–7.68 (m, 2H, H-2″′), 7.24–7.19 (m, 3H, H-5′ and H-3″′), 3.64 (t, *J* = 6.7 Hz, 2H, H-4), 2.42 (t, *J* = 6.7 Hz, 2H, H-3), 2.32 (s, 3H, Me-5″′), 1.96 (s, 3H, Me-1); ^13^C NMR (150 MHz, DMSO-*d*_6_): δ 129.3, 125.7, 102.8, 58.4, 41.5, 20.8, 16.9 (proportion of mixture of isomers *E*/*Z* could not be determined); ESI-MS: *m*/*z* calcd for [C_14_H_18_N_3_OS]^+^ 276.1092, found 276.1165.

#### General Procedure for the Synthesis of 2-Thiazolylhydrazines RVJ46, RVJ47, RVJ49, and RVJ62

A mixture of 1 equiv of thiosemicarbazide and 1 equiv of α-haloketone in methanol (5 mL) was stirred at 60 °C for 40 min when TLC analysis (eluent: *n*-hexane/EtOAc 3:7) revealed a total consumption of the starting material. The solvent was removed in a rotary evaporator under reduced pressure and the residue was quenched by adding 10 g of crushed ice, forming a precipitate. The solid material was filtrated in vacuum using a Büchner funnel and washed with cold distilled water, yielding a pure product.

##### 2-Hydrazineyl-4-phenylthiazole (RVJ46)

Following the general procedure described above, thiosemicarbazide (50.0 mg, 0.549 mmol) and 2-bromoacetophenone (109.2 mg, 0.549 mmol) were reacted to provide RVJ46 as a beige solid (30.6 mg, 29%). mp: 130.5–132.5 °C (lit.: 129 °C [ref]); ^1^H NMR (600 MHz, DMSO-*d*_6_): δ 8.58 (s, 1H, – NH–NH_2_), 7.80 (d, 2H, H-2′), 7.35 (t, *J* = 7.5 Hz, 2H, H-3′), 7.24 (t, *J* = 7.4 Hz, 1H, H-4′), 7.09 (s, 1H, H-5), 3.87 (s, 1H, – NH–NH_2_); ^13^C NMR (150 MHz, DMSO-*d*_6_): δ 175.9, 150.6, 135.2, 128.4, 127.1, 125.4, 102.0.

##### 2-Hydrazineyl-4-(4-methylphenyl)thiazole (RVJ47)

Following the general procedure described above, thiosemicarbazide (0.549 mmol) and 2-bromo-4′-methylacetophenone (0.549 mmol) were reacted to provide RVJ47 as a maroon solid (66.8 mg, 59%). mp: 120–122 °C; ^1^H NMR (600 MHz, DMSO-*d*_6_):): δ 7.72 (d, *J* = 7.8 Hz, 2H, H-2′), 7.18 (d, *J* = 7.8 Hz, 2H, H-3′), 7.14 (s, 1H, H-5), 2.30 (s, 3H, Me-5′); ^13^C NMR (150 MHz, DMSO-*d*_6_): δ 151.1, 136.7, 132.0, 129.6, 125.5, 102.4, 20.8.

##### 2-Hydrazineyl-4-(4-cyanophenyl)thiazole (RVJ49)

Following the general procedure described above, thiosemicarbazide (0.549 mmol) and 2-bromo-4′-cyanoacetophenone (0.549 mmol) were reacted to provide RVJ49 as a yellowish solid (50.3 mg, 42%). mp: 148–150 °C; ^1^H NMR (600 MHz, DMSO-*d*_6_): δ 8.77 (s, 1H, – NH–NH_2_), 7.97 (d, *J* = 8.4 Hz, 2H, H-2′), 7.82 (t, *J* = 8.4 Hz, 2H, H-3′), 7.43 (s, 1H, H-5), 4.67 (s, 1H, – NH–NH_2_); ^13^C NMR (150 MHz, DMSO-*d*_6_): δ 175.8, 148.9, 139.2, 132.5, 126.0, 119.1, 109.2, 106.0.

##### 2-Hydrazineyl-4-(4-fluorophenyl)thiazole (RVJ62)

Following the general procedure described above, thiosemicarbazide (50.0 mg, 0.549 mmol) and 2-bromo-4′-fluoroacetophenone (119.2 mg, 0.549 mmol) were reacted to provide **RVJ62** as a red solid (28.1 mg, 25%). mp: 122–124 °C (lit. 108 °C;^36^^1^H NMR (600 MHz, DMSO-*d*_6_): δ 8.34 (s, 1H, – NH–NH_2_), 7.97–7.89 (m, 2H, H-2′), 7.41–7.23 (m, 3H, H-5 and H-3′), 4.64 (s, 1H, – NH–NH_2_); ^13^C NMR (150 MHz, DMSO-*d*_6_): δ 164.0, 150.4, 129.6 (d, *J*_C,F_ = 9 Hz), 116.1 (d, *J*_C,F_ = 22.5 Hz), 115.7.

### Fungal Strains

Seven *C. gattii* strains (L27/01, 29/0893, 547 OTTI, 96806, 28/0893, 1962 and R265) and six *C. neoformans* strains (ATCC 24067, ATCC 28957, WM626, WM628, WM629, and H99) were tested. All strains were maintained at −80 °C and cultured on Sabouraud dextrose agar (SDA) medium at 37 °C for each experiment. In addition to these strains, we also used a *C. neoformans* H99 transcription factor knockout mutant collection. This collection has 322 mutant strains for 155 different transcription factors.^23^

### Experimental Design

To evaluate the *in vitro* antifungal activity of different thiazole derivatives against *Cryptococcus* spp., 30 substances were initially tested against two strains, *C. gatti* and *C. neoformans*.

A screening was initially performed with all compounds to evaluate their minimum inhibitory concentration (MIC) and minimum fungicidal concentration. Then, a cut was made for those that presented the least effective actions, and 12 compounds were further evaluated for their selectivity index. The compounds that presented the best activity and selectivity were then subjected to a growth curve experiment, in addition to the evaluation of ergosterol production by the yeasts after contact with the substances. Based on the results obtained, four substances were selected (RN86, RN88, RJ37, and RVJ42) and the remaining experiments were performed with these: a chemical-genetic screening using a collection of *C. neoformans* transcription factor mutants, a drug combination assay using amphotericin B and fluconazole, an evaluation of the influence of the derivatives on the resistance or susceptibility of the H99 lineage in the presence of cellular stressors, and transmigration assays to assess the ability of the substances to cross the BBB. Finally, assays with a murine model were performed to assess the probability of extending the survival of animals treated with thiazoles, in addition to evaluating the fungal load in the brain and lungs of animals.

### Toxicity against *In Vitro* Mammalian Cells

To evaluate the toxicity of the compounds on hCMEC/D3 cells (Merk-Millipore #SCC066), a viability test was performed using the MTT methodology.^38^ Cells were cultured in 96-well flat-bottom plates until they reached 90% confluency.^39^ Once reached, the cells were incubated for 48 h at 37 °C and 5% atmospheric CO_2_, together with a serial dilution of thiazole derivatives diluted in DMSO. Viability was measured by adding MTT and incubating for 4 h. After incubation, the MTT solution was carefully removed and DMSO was added to solubilize the formazan crystals. The plates were then gently shaken and incubated at room temperature for 5 min and read at an absorbance at 590 nm on a spectrophotometer. Toxicity assessment was carried out by calculating the selectivity index, this index is determined by the ratio between the cytotoxicity value and the minimum inhibitory concentration (MIC) value. Values higher than 10 were considered indicative of the absence of toxicity.^40^

### Thiazoles *In Vitro* Activity against *Cryptococcus*

Next, a screening of the compounds was performed by an assay to determine the MIC of each derivative against *C. neoformans* H99 and *C. gattii* R265, by broth microdilution methodology.^41^ Briefly, a fungal suspension containing (1–5) × 10^6^ cells/mL was diluted in RPMI 1640 plus 0.165 M MOPS, to achieve a suspension with 1 × 10^3^ to 5 × 10^3^ cells/mL. A serial dilution of the substances was prepared, and the final concentration ranged from 250 to 0.098 μg/mL. Fluconazole (0.125 to 64 μg/mL) and amphotericin B (0.03 to 8 μg/mL) were used as controls. The assay was performed in sterile microdilution plates with 96 flat-bottomed wells. The plates were incubated in a humid chamber at 37 °C for 72 h. Reading was carried out visually, and the MIC was considered as the total inhibition of growth compared to the growth of the control. For fluconazole, 50% growth inhibition was considered when compared with the control. After, 10 μL of the well from which the MIC value was obtained and the wells of 2 and 4 times the concentrations above were plated in a drop on SDA and were incubated in a humid chamber at 37 °C for 72 h to obtain thee minimal fungicide concentration (MFC).^42^

### Fungicidal Kinetics

After the MIC of the thiazole derivatives was determined, a growth curve assay was performed with the best derivatives so far (RN86, RN88, RJ37, and RVJ42) to evaluate the influence of the compounds on yeast growth. Initially, plates were prepared containing thiazole dilutions in concentrations equal to the MIC, 2 × MIC, and 4 × MIC concentrations in addition to a positive growth control with RPMI medium plus fungal inoculum. The plates were incubated at 37 °C, and plating was performed on SDA solid medium after 3, 6, 24, and 48 h. ASD plates were incubated at 37 °C, and colony-forming units (CFU) were quantified after 72 h.^43^

### Chemical-Genetic Screening

A 322 mutant library was used in this assay,^23^ kindly provided by Dr. Goldman (University of São Paulo/Brazil). Each transcription factor mutant strain was grown in SDA medium at 28 °C for 48 h, and then a fungal suspension containing 1–5 × 10^3^ cells/mL of each mutant was prepared and added into flat-bottomed 96-well plates containing concentrations ranging from 4 times the MIC value to MIC/4 of thiazole derivatives for the H99 wildtype strain. These plates were incubated in a humid chamber at 28 °C for 72 h and then followed for visual assessment of growth.

*In silico* analysis was carried out for the genes for which the knockout strains showed resistance (MIC value equal to or greater than 4× the MIC of the wild-type strain) or sensitivity (MIC value equal to or less than 4× the MIC of the wild strain) to the compounds. The annotation of which gene and their function were retrieved from the public database FungiDB (fungidb.org). Then, functional enrichment of these genes was performed based on gene ontology, molecular function, and biological process.^44^

### Investigation of the Mechanism of Action of Thiazoles

#### Stress Susceptibility Tests

After verifying the susceptibility of *C. neoformans* to the thiazoles, the next step was to investigate the possible cellular changes induced by these substances.^44^ For this, *C. neoformans* H99 was previously cultivated in SDA for 72 h at 37 °C, and a fungal suspension of 2 × 10^4^ cells/mL was prepared in RPMI 1640 medium with 0.165 M MOPS and the thiazole derivatives in subinhibitory concentrations (half of the MIC) and incubated at 37 °C for 48 h. After exposure to the derivatives, a new inoculum containing 1 × 10^6^ cells/mL and four 1:10 serial dilutions of each treatment was prepared, and 5 μL of each dilution was dispensed in SDA supplemented with different cellular stressors: 0.5% Congo red (cell wall stress), 0.025% triton-100 (cell wall and membrane stress), 1.5 M sodium chloride (osmotic stress), 1.5 M potassium chloride (osmotic stress), and 0.03% sodium dodecyl sulfate (SDS–membrane and cell wall stress).^23^ In addition, the inoculum was used to evaluate melanin production in solid minimal medium (MM) (15 mM glucose, 10 mM MgSO_4_, 29.4 mM KHPO_4_, 13 mM glycine, and 18 g/L agar, pH 5.5) supplemented with 1 mM L-dopa.^44^ Plates were incubated at 37 °C for 72 h and subsequently photographed and analyzed.

### Interaction between Thiazoles and Clinical Antifungals

Thiazole derivatives were tested for their activity in combination with fluconazole and amphotericin B by the checkerboard method.^45^ Ten serial dilutions of thiazoles (25.0 to 0.05 μg/mL) and seven dilutions of antifungals were prepared (fluconazole from 64.0 to 1.0 μg/mL and amphotericin B from 16.0 to 0.25 μg/mL). Then, 50 μL aliquots of each derivative were added to the wells of a 96-well plate in a vertical orientation, and 50 μL aliquots of fluconazole or amphotericin B were added in a horizontal orientation so that the plate had all the different possible combinations of concentrations of the two drugs.^45^ Wells with different dilutions of the drugs alone were used as controls. Along with the drug dilutions, a fungal suspension containing 1 × 10^3^ to 5 × 10^3^ cells/mL was added, and plates were incubated at 37 °C for 72 h. The interaction of drugs with antifungals was calculated according to the ZIP reference model, using the SynergyFinder 3.0 platform.^46^

### Quantitation of Ergosterol

An inoculum of 1 × 10^8^ cells/mL of *C. gattii* R265 and *C. neoformans* H99 was prepared and incubated overnight with inhibitory concentrations of fluconazole and thiazole derivatives. For lipid extraction, 25% ethanolic potassium hydroxide solution was added to each cell mass, followed by intense agitation in a vortex shaker.^31^ Subsequently, the tubes were incubated in a water bath at 85 °C for 1 h and then cooled to room temperature. A mixture of 3 mL of *n*-heptane (Sigma-Aldrich) and 1 mL of sterilized distilled water was added to the mixture, followed by stirring for 3 min on a vortex mixer. The supernatants were placed in 96-well flat-bottom plates and read on a spectrophotometer at 282 nm. An ergosterol standard curve (Sigma-Aldrich) was performed and used for ergosterol quantification.

### *ExVivo* Studies

#### Fungal Transcytosis Assay in an *In Vitro* Blood-Brain Barrier (BBB) Model

hCMEC/D3 cells were cultured in the upper part of a Transwell apparatus with a collagen-covered 8 μm pore membrane using approximately 5 × 10^4^ cells per cm^2^ until 100% confluency.^39^ Then, 1 × 10^6^ fungal cells in RPMI-1640 medium were added to the apical part of the chamber. Along with the yeast, inhibitory concentrations of the compounds were added to the apical or the basal part of the apparatus, and they were incubated at 37 °C/5% CO_2_ for 24 h. After this period, 100 μL of the lower part was collected, plated on SDA, and incubated for 48 h at 37 °C for CFU (colony forming unit) counting.

### Derivatives Transmigration across the *In Vitro* BBB Model: Development and Validation of HPLC/DAD Bioanalytical Methods for Quantification of Thiazoles

To study the ability of thiazole to cross the BBB, we quantified the concentration of the substances in the basal part of the transwell after 24 h of its addition to the apical part. Two different protocols were used to detect the molecules: for the thiazoles, RJ37 and RVJ42 used the Zorbax Eclipse XDB C18 column and a mobile phase buffer containing 2 mM ammonium acetate buffer and 0.1% formic acid, pH 2.5; and for RN86 and RN88 used a column of phenyl and mobile phase buffer of 7.2 mM sodium acetate and 1% acetic acid, pH 5.7. Both methods used an injection volume of 10 μL, an injection flow of 1 mL/min, a column oven temperature of 40 °C, and a detector wavelength of 260 nm. To perform the chromatographic method, standard solutions of 1 mg/mL were prepared for each compound on the day of analysis, and individual runs were performed to observe the detection time of each thiazole derivative.

A calibration curve was constructed using five different concentrations of each of the molecules independently. To prepare the concentrations, the stock solution previously prepared for each of the compounds was used as a base and the new concentrations were prepared by diluting with Hank’s balanced solution without calcium and without magnesium (5.33 mM KCl, KH_2_PO_4_ 0.44 mM, 138 mM NaCl, 4 mM NaHCO_3_, 0.3 mM Na_2_HPO_4_, and 5.6 mM glucose), and their concentrations were 0.5, 1, 5, 10, and 20 μg/mL. The calibration curve was constructed by determining the equation of the straight line generated from the five points, and through this, it was possible to calculate unknown concentrations in a sample through the generated signal.

### *InVitro* Permeability Study in HCMEC/D3

After the development and validation, we carried out the determination of the thiazole concentration in the *in vitro* BBB model. After the BBB formation and confluence in the transwell, the culture medium from the apical part was aspirated and washed with Hank’s balanced solution without calcium and magnesium. Then, 200 μL of the compounds at concentrations of 20 and 200 μg/mL, diluted in Hank’s balanced solution, were added to these wells. The plates containing the transwells were incubated at 37 °C/5% CO_2_ for 24 h and then proceeded for bioanalytical quantification using the best conditions described above.

### *InVivo* Studies

#### Ethics

This study was approved by the Ethics Committee in the Use of Animals (CEUA) of the Universidade Federal de Minas Gerais (protocol 279/2023). We followed the Brazilian Society of Zootechnics/Brazilian College of Animal Experimentation guidelines and Federal Law 11.794/2008. Water and food were provided *ad libitum,* and light/dark cycles were maintained. All efforts to minimize the suffering of the animals were carried out.

### Evaluation of Survival in a Murine Model of *C. neoformans* Infection

For infection, C57/BL6 mice were anesthetized with ketamine and xylazine (80 and 15 mg/kg, respectively). Intratracheal infection was carried out by a small incision in the skin, close to the thyroid, and after separation of the tissue layers, the trachea was exposed and inoculated with 1 × 10^5^ cells in 30 μL, and then the incision was sutured. Mice were divided into seven groups (*n* = 6 animals/group), according to the treatments, starting 24 h after infection (intraperitoneally injections once daily): (1) RN86, (2) RN88, (3) RJ37, (4) RVJ42 (10 mg/kg),^47^ (5) AMB (0.5 mg/kg),^44^ (6) nontreated (NT), and (7) noninfected (NI). Mice were monitored daily, and animals showing weight loss greater than 20%, tremors, or immobility were humanely euthanized in accordance with CONCEA (National Council for Control of Animal Experimentation) standards.

### Determination of Fungal Burden in the Brain and Lung

After the survival evaluation, two new tests were carried out to analyze the fungal load after infection and treatment of the animals. Infection and treatment were carried out as previously described, and the animals (*n* = 6 animals/group) were euthanized 10 days postinfection, and brain and lung were harvested to determine fungal burden. Then, intracranial infection was performed to simulate the treatment of an already consolidated brain infection, for that, mice were anesthetized, and 1 × 10^3^ fungal cells in a volume of 5 μL were inserted directly into the brain tissue through the skull, using a syringe. After 5 days, animals were euthanized, and the brain was collected. In both experiments, the animals were treated with the same doses of the compounds from the previous experiment. After homogenizing the organs in PBS, 50 μL of each lung and 200 μL of each brain were plated on SDA and incubated at 37 °C for 48 h. Subsequently, colonies were visually counted, and the number of CFU/g of each organ was determined.

### Statistical Analysis

Statistical analyses were performed using GraphPad Prism version 9.5 (GraphPad Inc., San Diego, CA, USA), with *p* < 0.05 considered to be significant. Time–kill curves were analyzed by the area under the curve. Ergosterol content and CFU were analyzed by analysis of variance (ANOVA), followed by the nonparametric Friedman post-test. The survival curve was plotted by the Kaplan–Meier method, and the results were analyzed using the log-rank test.

## Supporting Information Available

^1^H and ^13^C nuclear magnetic resonance (NMR) spectra (Figures S1–S12); HRMS (ESI) spectrum (Figure S13); and fungal kinetics (Figures S14 and S15) (PDF)
