# Supplementary material for: Thiazole Derivatives as Promising Candidates for Cryptococcosis Therapy
Source: ACS Infect Dis. 2025 Feb 7;11(3):639–52. doi: 10.1021/acsinfecdis.4c00732 (PMC11915371; doi:10.1021/acsinfecdis.4c00732)

SUPPORTING INFORMATION

Thiazole Derivatives as Promising Candidates for Cryptococcosis Therapy

Victor Augusto Teixeira Leocádio[1]; Isabela L. Miranda[1]; Martha H. C. Magalhães[1]; Valtair Severino dos Santos Júnior[2]; José Eduardo Goncalves[2]; Renata Barbosa Oliveira[2]; Vinicius Gonçalves Maltarollo[2]; Rafael Wesley Bastos[3,4]; Gustavo Goldman[5]; Susana Johann[1]; Nalu Teixeira de Aguiar Peres[1]; Daniel de Assis Santos[1, 4*]

[1] Universidade Federal de Minas Gerais, Departamento de Microbiologia, Belo Horizonte, Brazil.

[2] Universidade Federal de Minas Gerais, Departamento de Produtos Farmacêuticos, Faculdade de Farmácia, Belo Horizonte, Brazil.

[3] Universidade Federal do Rio Grande do Norte, Centro de Biociências, Natal, Brazil.

[4] National Institute of Science and Technology in Human Pathogenic Fungi, Brazil.

[5] Universidade de São Paulo, Faculdade de Ciências Farmacêuticas de Ribeirão Preto, Ribeirão Preto, Brazil.

***Corresponding author:** Daniel Assis Santos, Departamento de Microbiologia, Instituto de Ciências Biológicas, Universidade Federal de Minas Gerais, Av. Antônio Carlos, 6627, Pampulha, Belo Horizonte, Minas Gerais, Brazil, 31270-901. E-mail: das@ufmg.br or dasufmg@gmail.com; Tel: +55 31 3409 2758; Fax: +55 31 3409 2733.

CONTENTS

^{1}H and ^{13}C Nuclear Magnetic Resonance (NMR) spectra (Figures S1-S12)

HRMS (ESI) spectrum (Figure S13)

Fungal kinects (Figures S14 and S15)

1. ^{1}H and ^{13}C Nuclear Magnetic Resonance (NMR) spectra

Figure S1. ^{1}H NMR spectra (400 MHz, DMSO-d_6) of compound **RVJ39**.

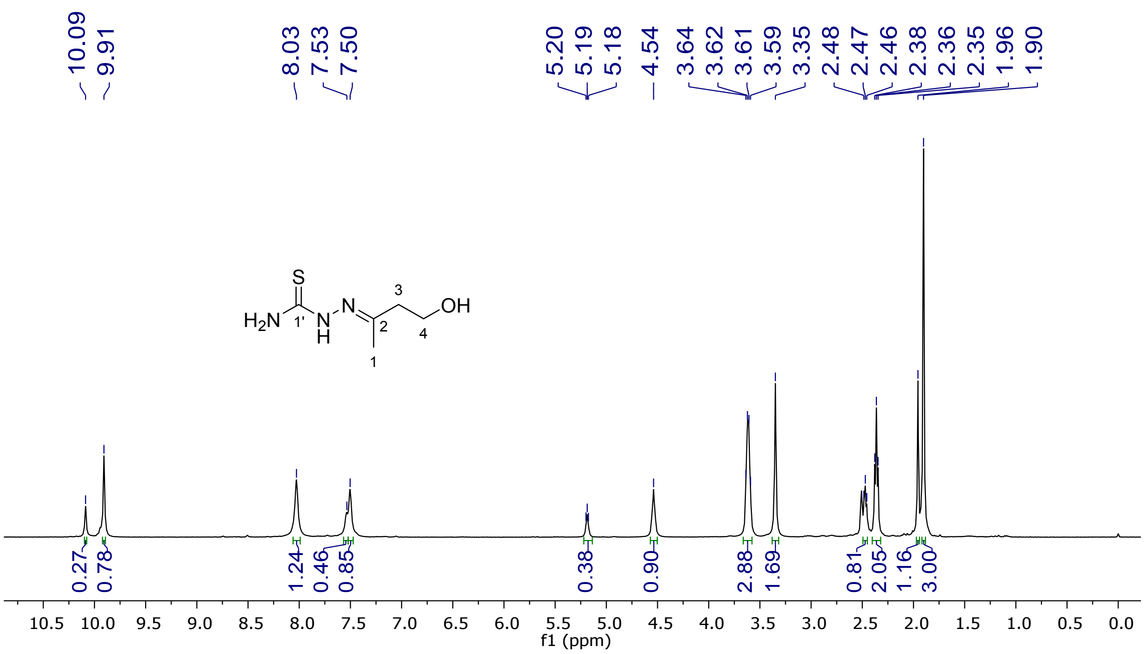

Figure S2. ^{13}C and DEPT-135 spectra (100 MHz, DMSO-d_6) of compound **TS3**.

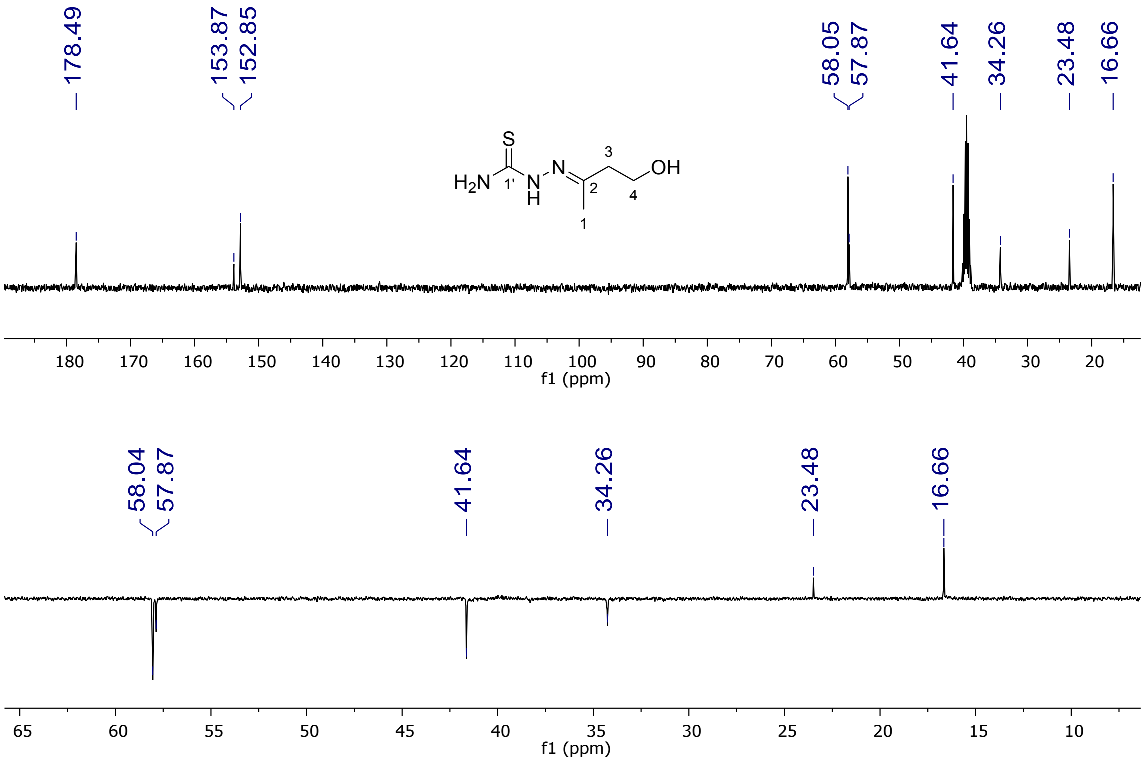

Figure S3. ^{1}H NMR spectra (600 MHz, DMSO-d_6) of compound **RVJ45**.

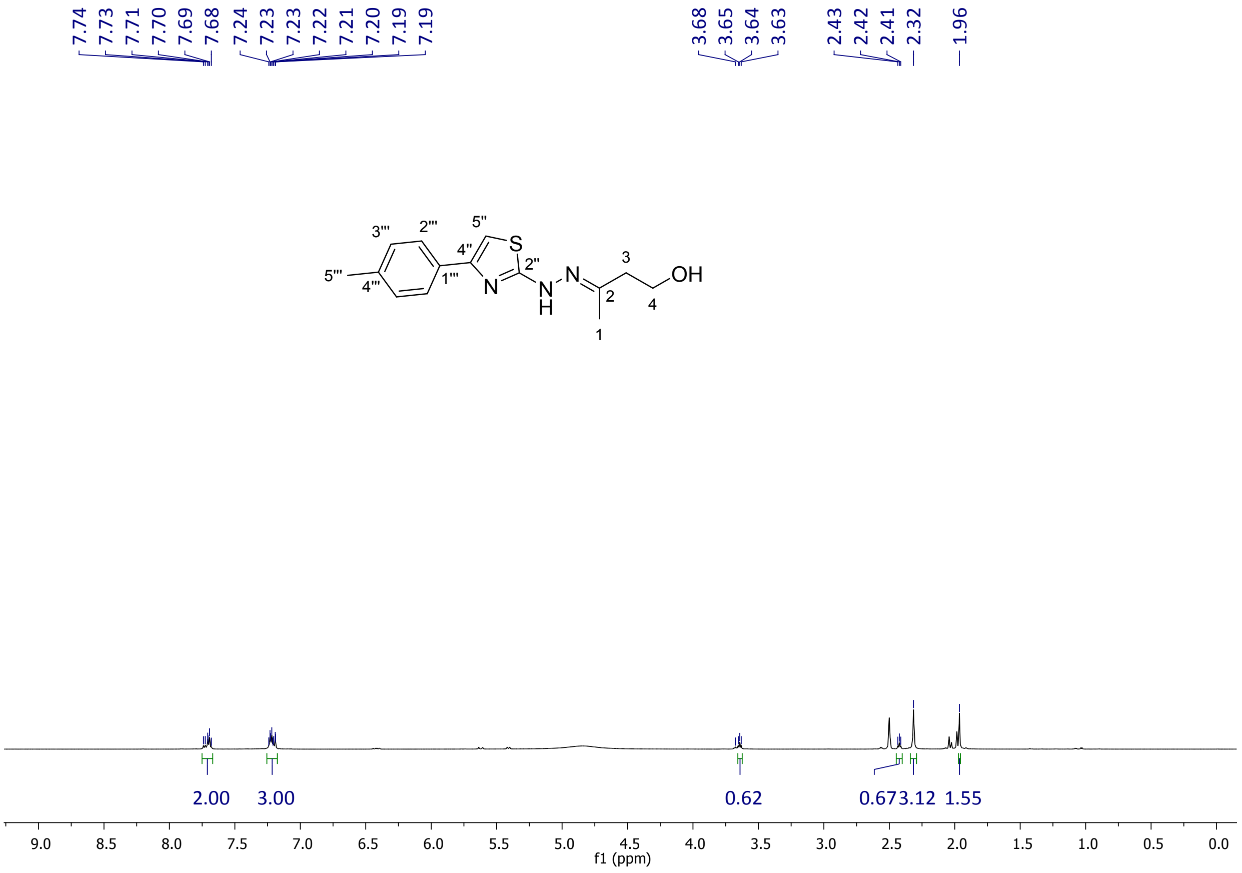

Figure S4. ^{13}C NMR and DEPT-135 spectra (150 MHz, DMSO-d_6) of compound **RVJ45**.

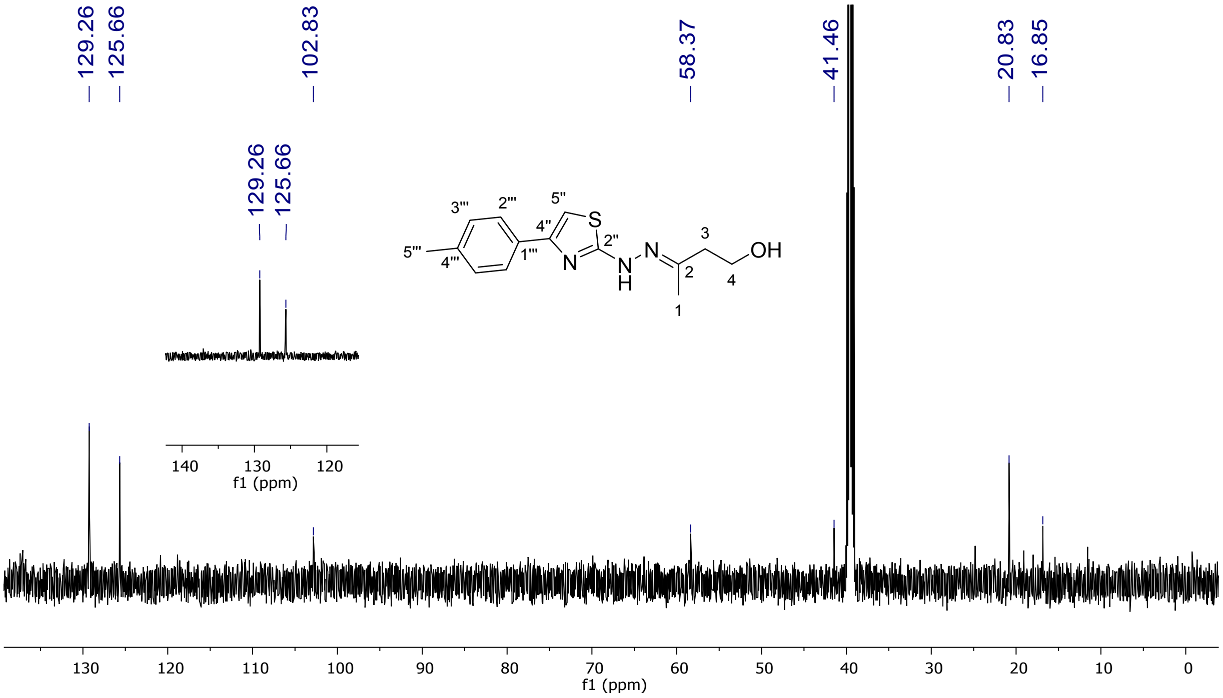

Figure S5. ^{1}H NMR spectra (600 MHz, DMSO-d_6) of compound **RVJ46**.

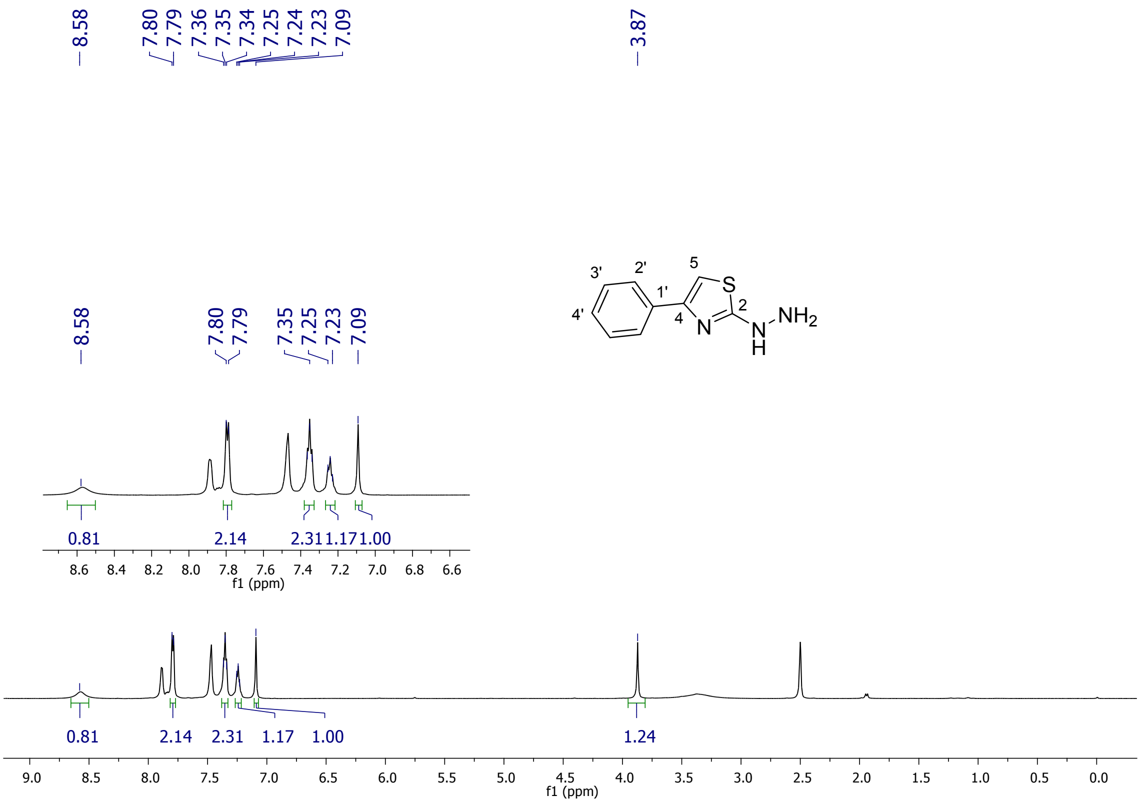

Figure S6. ^{13}C NMR and DEPT-135 spectra (150 MHz, DMSO-d_6) of compound **RVJ46**.

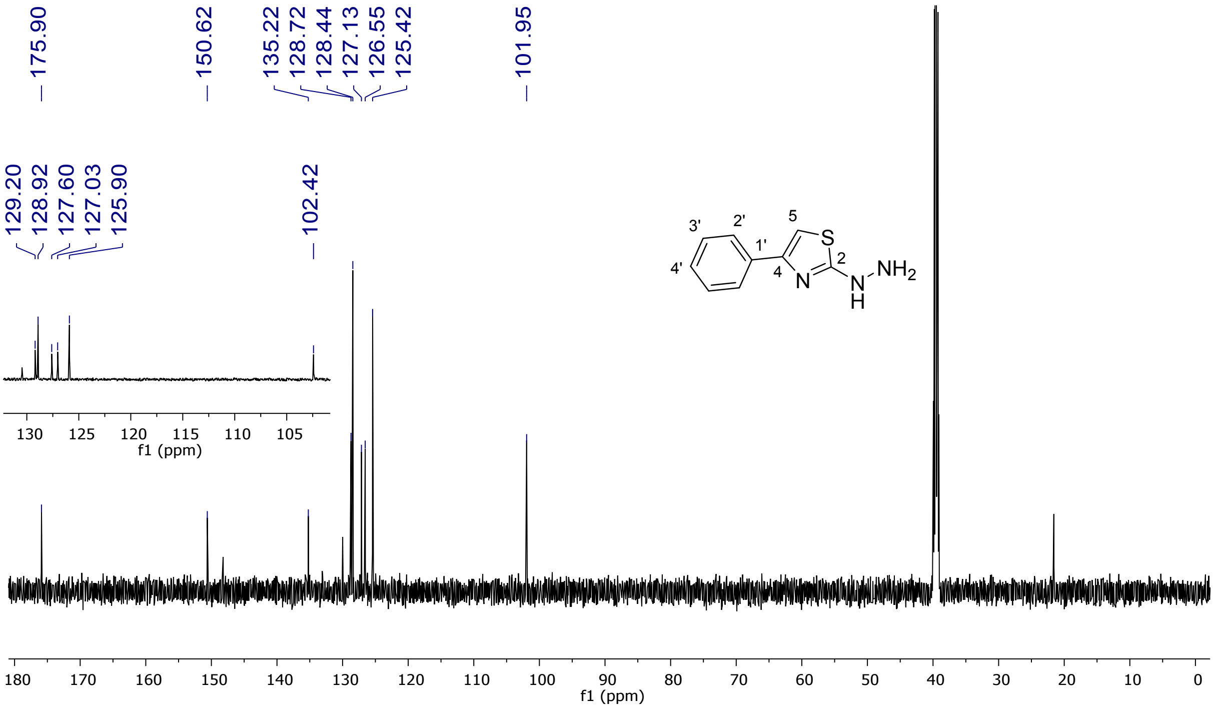

Figure S7. ^{1}H NMR spectra (600 MHz, DMSO-d_6) of compound **RVJ47**

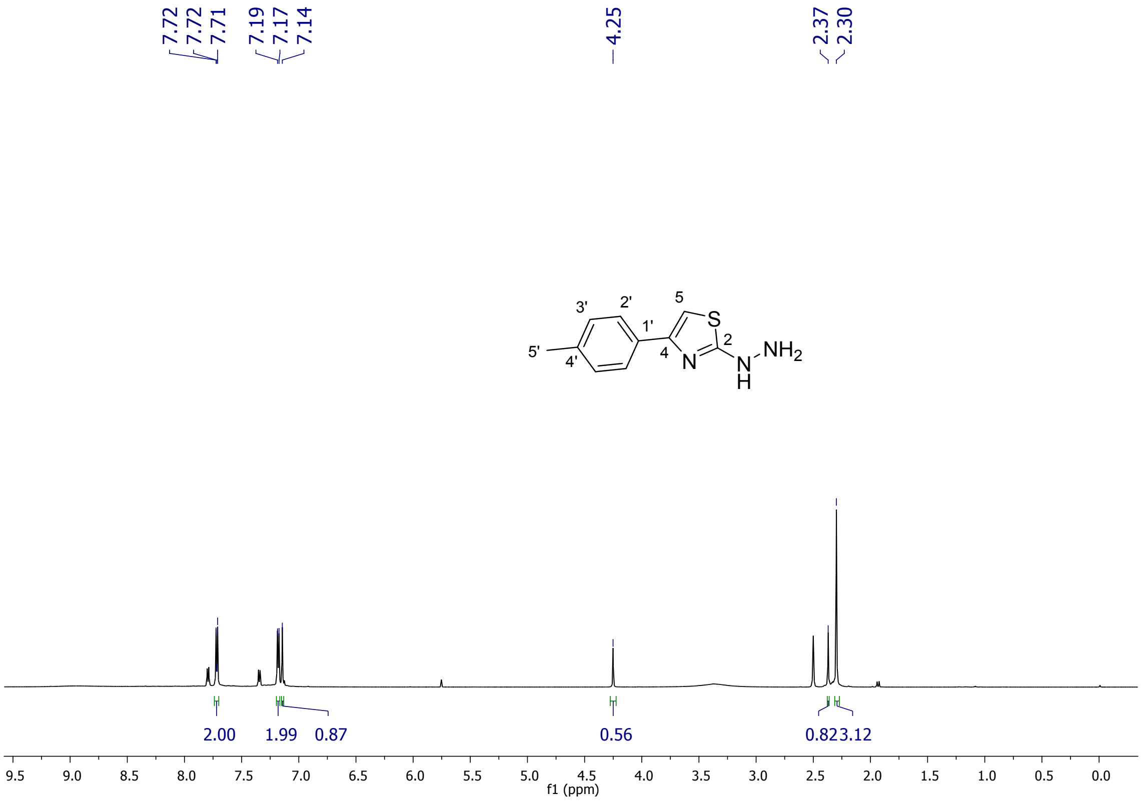

Figure S8. ^{13}C NMR and DEPT-135 spectra (150 MHz, DMSO-d_6) of compound **RVJ47**.

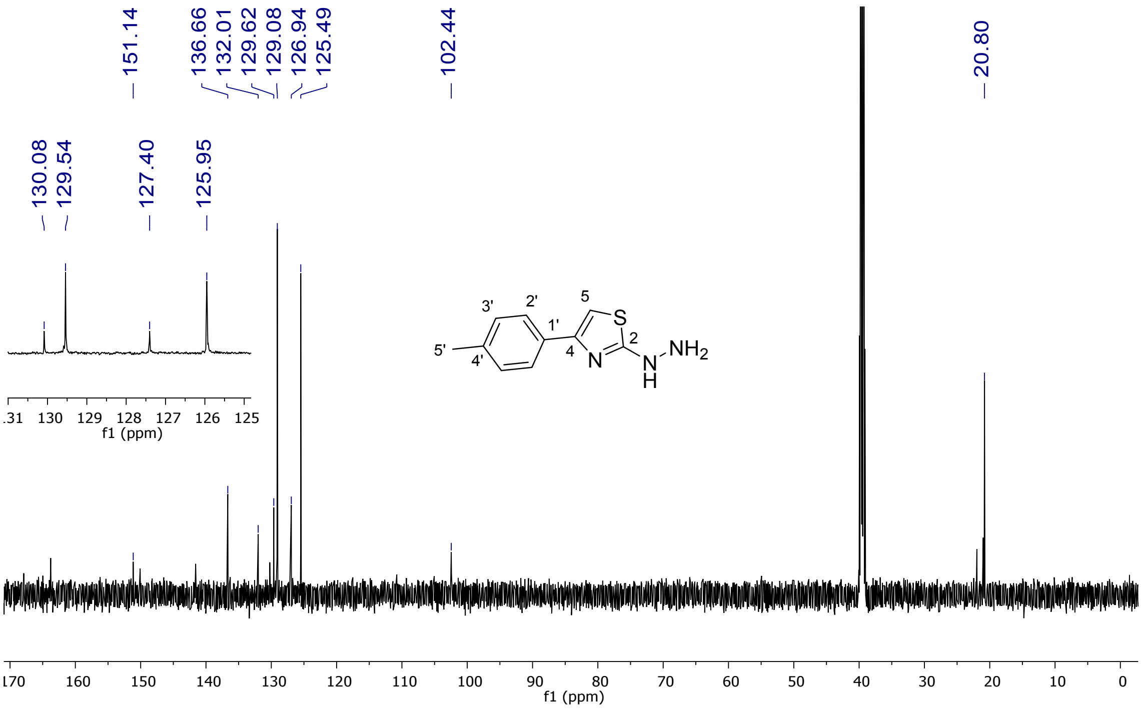

Figure S9. ^{1}H NMR spectra (600 MHz, DMSO-d_6) of compound **RVJ49**.

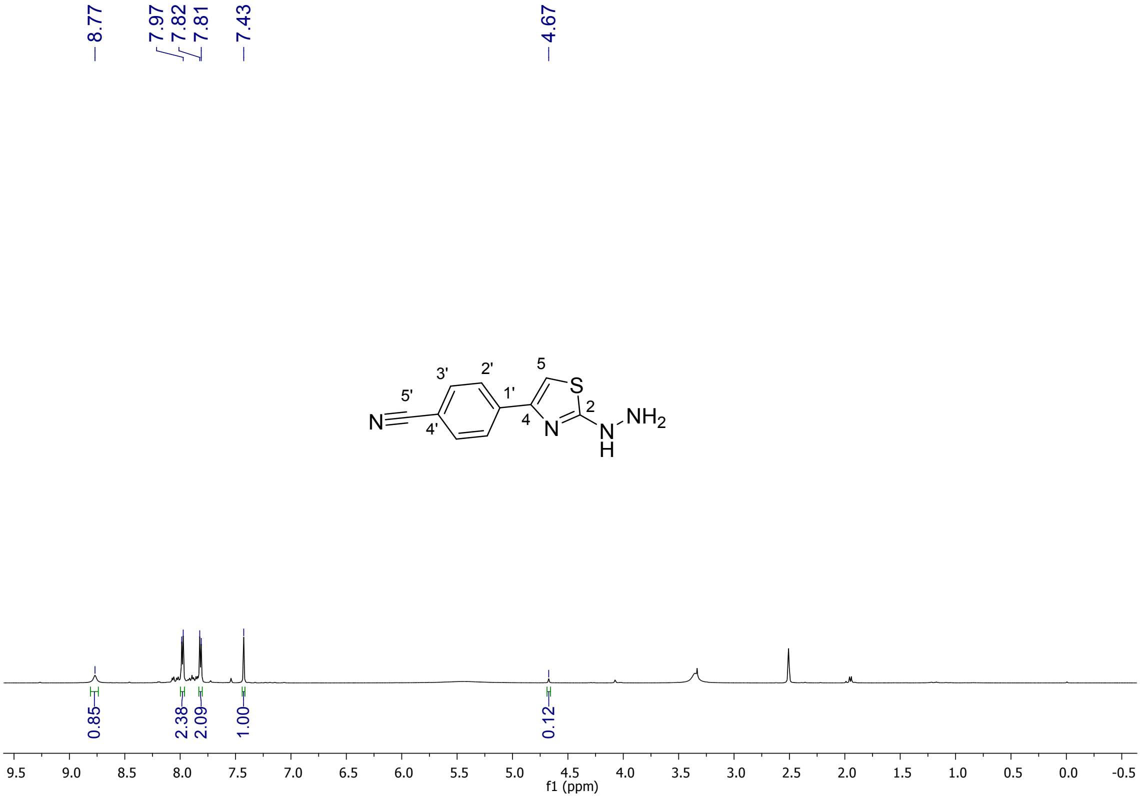

Figure S10. ^{13}C NMR and DEPT-135 spectra (150 MHz, DMSO-d_6) of compound **RVJ49**.

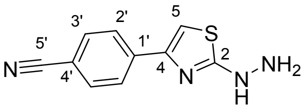

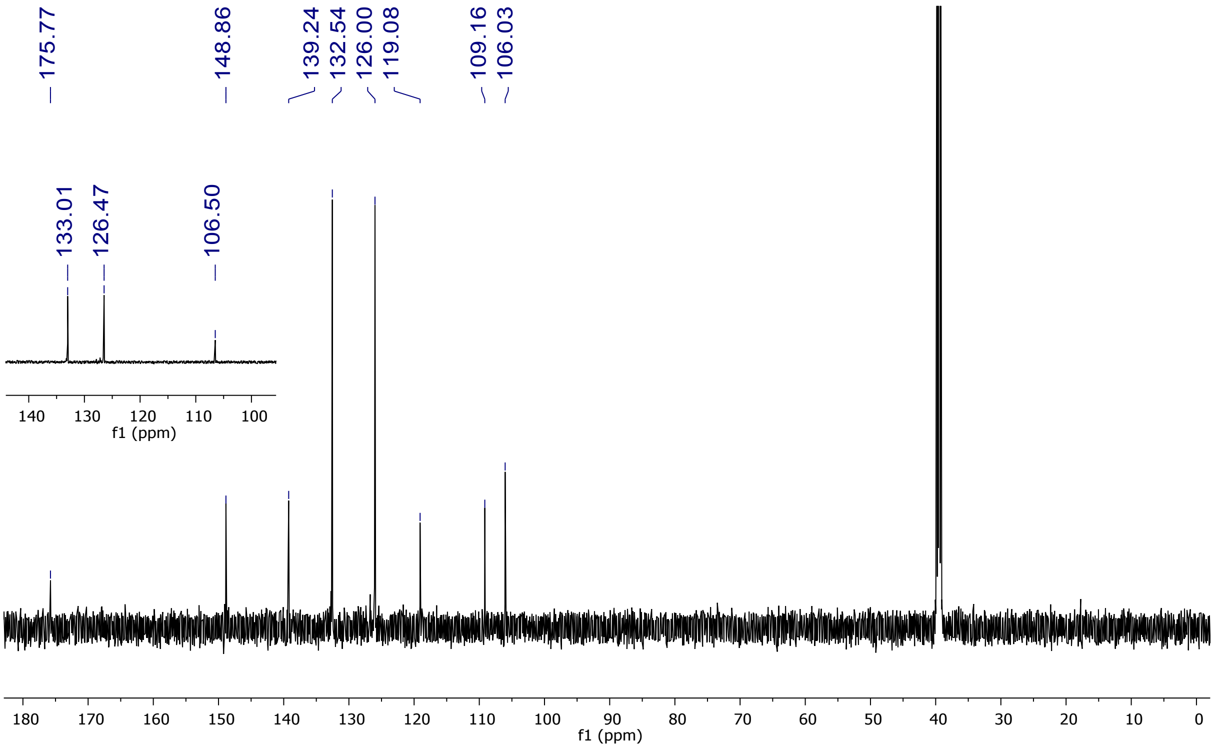

Figure S11. ^{1}H NMR spectra (600 MHz, DMSO-d_6) of compound **RVJ62**.

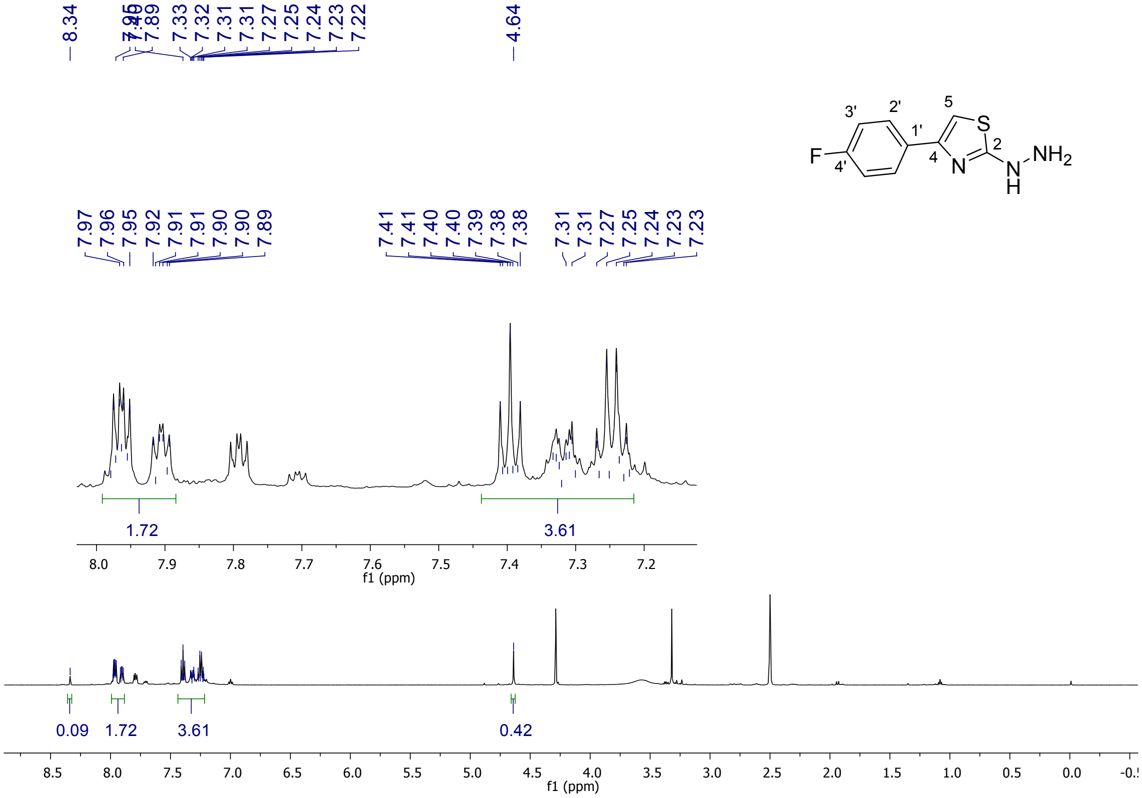

Figure S12. ^{13}C NMR and DEPT-135 spectra (150 MHz, DMSO-d_6) of compound **RVJ62**.

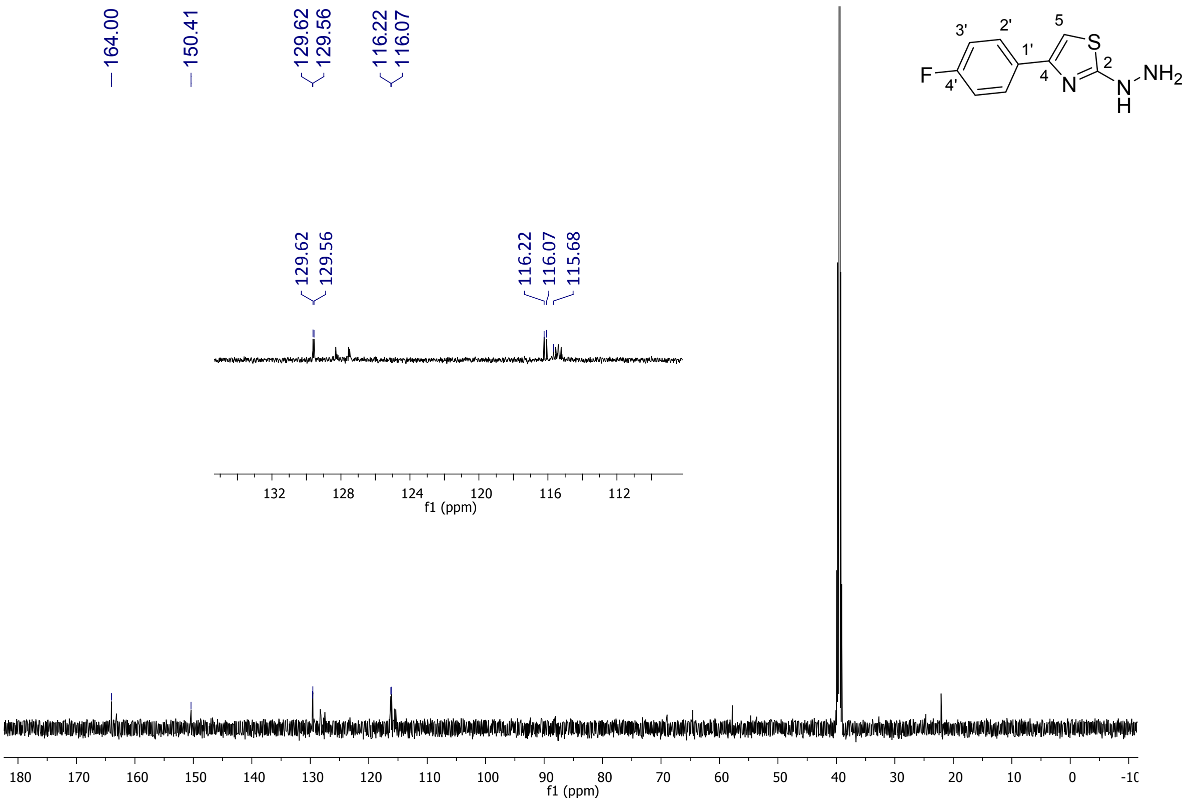

2. HRMS (ESI) spectrum

Figure S13. HRMS/MS (ESI) of compound **RVJ45**.

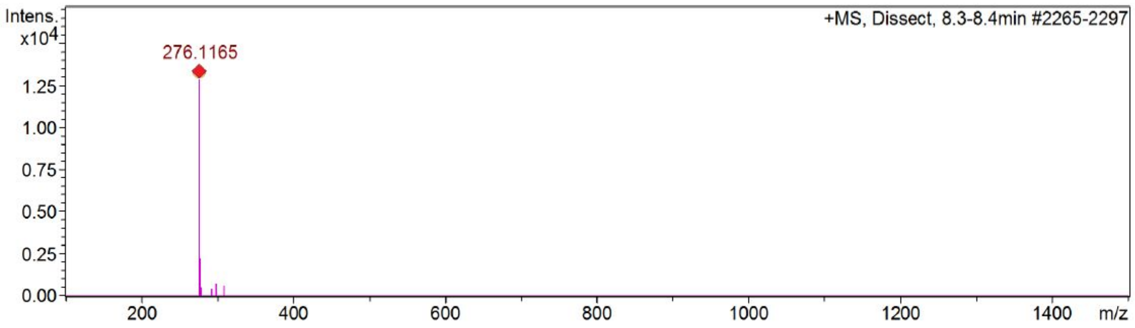

Figure S14. Effect of MIC concentration on the growth of *Cryptococcus neoformans* H99 strain. Statistical analysis: Area under the curve; one-way ANOVA followed by Tukey post-test. * $p < 0.05$ compared to growth control. MIC: Minimum Inhibitory Concentration.

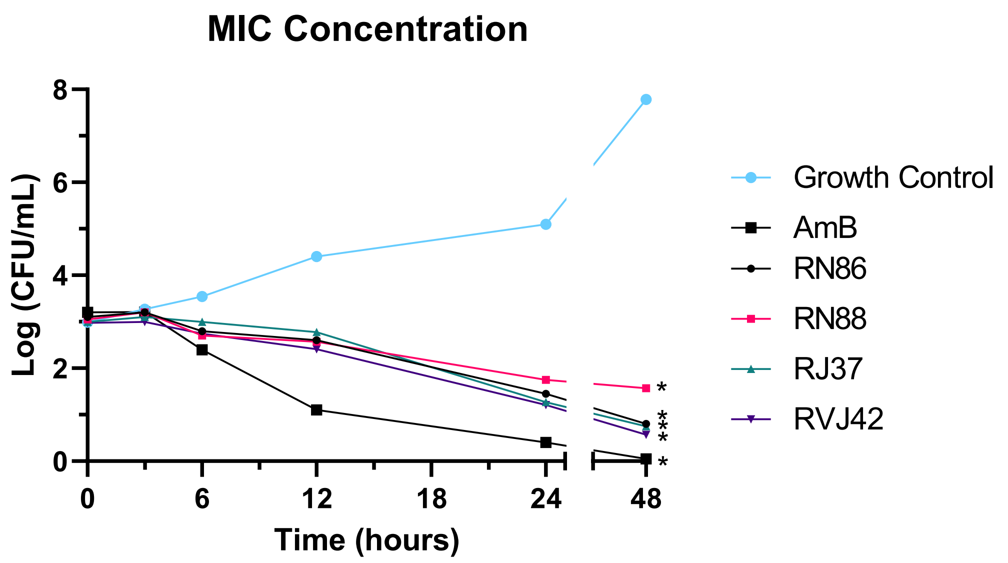

Figure S15. Effect of 2XMIC concentration on the growth of *Cryptococcus neoformans* H99 strain. Statistical analysis: Area under the curve; one-way ANOVA followed by Tukey post-test. * $p < 0.05$ compared to growth control. MIC: Minimum Inhibitory Concentration.

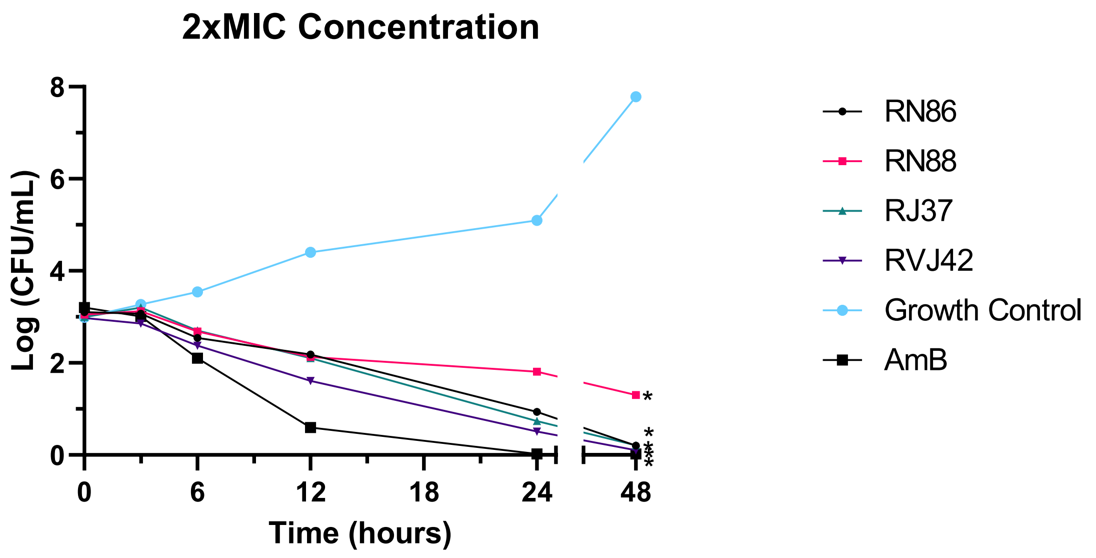

Supplement: Supplementary file 1 — id4c00732_si_001.pdf [file id4c00732_si_001.pdf]
